# A Novel Enterococcus faecalis Heme Transport Regulator (FhtR) Senses Host Heme To Control Its Intracellular Homeostasis

**DOI:** 10.1128/mBio.03392-20

**Published:** 2021-02-02

**Authors:** Vincent Saillant, Damien Lipuma, Emeline Ostyn, Laetitia Joubert, Alain Boussac, Hugo Guerin, Géraldine Brandelet, Pascal Arnoux, Delphine Lechardeur

**Affiliations:** a Micalis Institute, INRAE, AgroParisTech, Université Paris-Saclay, Jouy-en-Josas, France; b I2BC, CNRS UMR 9198, CEA Saclay, Gif-sur-Yvette, France; c Aix Marseille Université, CEA, CNRS, BIAM, Saint Paul-Lez-Durance, France; University of Minnesota Medical School

**Keywords:** *Enterococcus faecalis*, heme homeostasis, heme transport, microbiota, stress adaptation, transcriptional regulation

## Abstract

Enterococcus faecalis is a commensal Gram-positive pathogen found in the intestines of mammals and is also a leading cause of severe infections occurring mainly among antibiotic-treated dysbiotic hospitalized patients. Like most intestinal bacteria, E. faecalis does not synthesize heme (in this report, heme refers to iron protoporphyrin IX regardless of the iron redox state). Nevertheless, environmental heme can improve E. faecalis fitness by activating respiration metabolism and a catalase that limits hydrogen peroxide stress. Since free heme also generates toxicity, its intracellular levels need to be strictly controlled. Here, we describe a unique transcriptional regulator, FhtR (named FhtR for faecalis heme transport regulator), which manages heme homeostasis by controlling an HrtBA-like efflux pump (named HrtBA*_Ef_* for the HrtBA from E. faecalis). We show that FhtR, by managing intracellular heme concentration, regulates the functional expression of the heme-dependent catalase A (KatA), thus participating in heme detoxification. The biochemical features of FhtR binding to DNA, and its interaction with heme that induces efflux, are characterized. The FhtR-HrtBA*_Ef_* system is shown to be relevant in a mouse intestinal model. We further show that FhtR senses heme from blood and hemoglobin but also from crossfeeding by Escherichia coli. These findings bring to light the central role of heme sensing by FhtR in response to heme fluctuations within the gastrointestinal tract, which allow this pathogen to limit heme toxicity while ensuring expression of an oxidative defense system.

## Abstract (precis)

Enterococcus faecalis, a normal and harmless colonizer of the human intestinal flora can cause severe infectious diseases in immunocompromised patients, particularly those that have been heavily treated with antibiotics. Therefore, it is important to understand the factors that promote its resistance and its virulence. E. faecalis, which cannot synthesize heme, an essential but toxic metabolite, needs to scavenge this molecule from the host to respire and fight stress generated by oxidants.

## INTRODUCTION

Enterococcus faecalis is a commensal inhabitant of the gastrointestinal tract (GIT) and a subdominant species in the core intestinal microbiota of healthy humans and other mammals (1). This lactic acid bacterium is also a major opportunistic pathogen that causes a large number of nosocomial infections such as endocarditis, bacteremia, urinary tract infections, or meningitis (2). In recent decades, E. faecalis has emerged as a leading cause of enterococcal infections, and it is the third most frequent source of hospital-acquired nosocomial infections (3). E. faecalis is thus considered a major public health threat due to its intrinsic resistance to antibiotics and the emergence of multidrug-resistant isolates (3). Selective outgrowth of enterococci following intestinal dysbiosis is frequent, regardless of whether it results from antibiotic treatment, intestinal inflammation, or infection (4). In addition to intrinsic and acquired antibiotic resistances, E. faecalis is resistant to other antimicrobial factors, such as bile, and tolerates a wide variety of stress factors such as temperature, pH, oxygen tension, or oxidation (1).

For most living organisms, heme (iron porphyrin) (in this report, heme refers to iron protoporphyrin IX regardless of the iron redox state, whereas hemin refers to ferric iron protoporphyrin IX) is an essential cofactor of enzymes such as cytochromes, catalases, or peroxidases (5). The importance of heme resides in the unique properties of its iron center, including the capacity to undergo electron transfer, to perform acid-base reactions and to interact with various coordinating ligands (6). Paradoxically, the high potential redox of heme iron catalyzes the production of reactive oxygen species (ROS). Oxidative stress generated by heme together with its accumulation in membranes explains its toxicity (7–9). Most bacteria carry the enzymatic machinery for endogenous heme synthesis. However, numerous bacteria lack some or all the enzymes needed for autosynthesis but still require this molecule for their metabolism (5). Interestingly, E. faecalis, like the majority of species constituting the ga`strointestinal microbiota, cannot synthesize heme (10, 11). When heme is added to an aerated culture, E. faecalis activates a terminal cytochrome *bd* oxidase, causing a shift from fermentation to an energetically favorable respiratory metabolism (11, 12). E. faecalis, unlike other *Firmicutes* that cannot synthesize heme, also carries a gene that encodes a heme catalase (KatA; EC 1.11.1.6), limiting hydrogen peroxide stress when heme is available (10). Both activities contribute to the virulence of several Gram-positive pathogens (13, 14). Although the importance of heme as a cofactor for numerous cellular functions is established (5, 15), the mechanisms governing exogenous heme internalization and secretion that contribute to heme homeostasis vary among bacteria and are poorly understood. However, heme homeostasis must be strictly regulated in all bacteria to avoid toxicity (6, 8). Heme efflux is a documented defense mechanism against heme toxicity in some *Firmicutes*. (i) The Pef regulon comprises two multidrug resistance efflux pumps and a MarR-type heme-responsive regulator in Streptococcus agalactiae (16). (ii) The HatRT system involves a TetR family heme binding transcriptional regulator (HatR) and a major facilitator superfamily heme transporter (HatT) in Clostridium difficile (17). (iii) Heme homeostasis in several Gram-positive bacteria relies on HrtBA (heme-regulated transport) proteins, an ABC transporter, which promotes heme efflux (14, 18–20). Expression of *hrtBA* is controlled by *hssRS* genes, encoding a two-component heme sensor and response regulator in numerous Gram-positive pathogens, including S. agalactiae, Staphylococcus aureus, and Bacillus anthracis (14, 19–22). In contrast, the food bacterium Lactococcus lactis regulates HrtBA expression via the TetR family heme sensor HrtR (19). To date, the mechanisms involved in E. faecalis management of environmental heme are unknown.

In this work, we describe the mechanism by which a novel E. faecalis TetR regulator, called FhtR (for faecalis heme transport regulator), induces expression of HrtBA*_Ef_* (named HrtBA*_Ef_* for the HrtBA from E. faecalis), a conserved heme efflux transporter. We show that FhtR binds intracellular heme, resulting in derepression and increased transcription of *hrtBA_Ef_*. Heme iron coordination specifies FhtR as a heme sensor, and a critical role for the tyrosine 132 was defined. Our results also establish this system as a master mechanism of control of intracellular heme availability as shown by its requirement for the expression of the heme-dependent E. faecalis KatA. Finally, the relevance of the FhtR system to E. faecalis is shown in a mouse intestine model, suggesting the importance of FhtR for E. faecalis adaptation in the GIT. Our conclusions lead to a new picture of heme homeostasis in E. faecalis.

## RESULTS

### The conserved heme efflux transporter HrtBA*_Ef_* is functional in E. faecalis.

**FIG 1:**
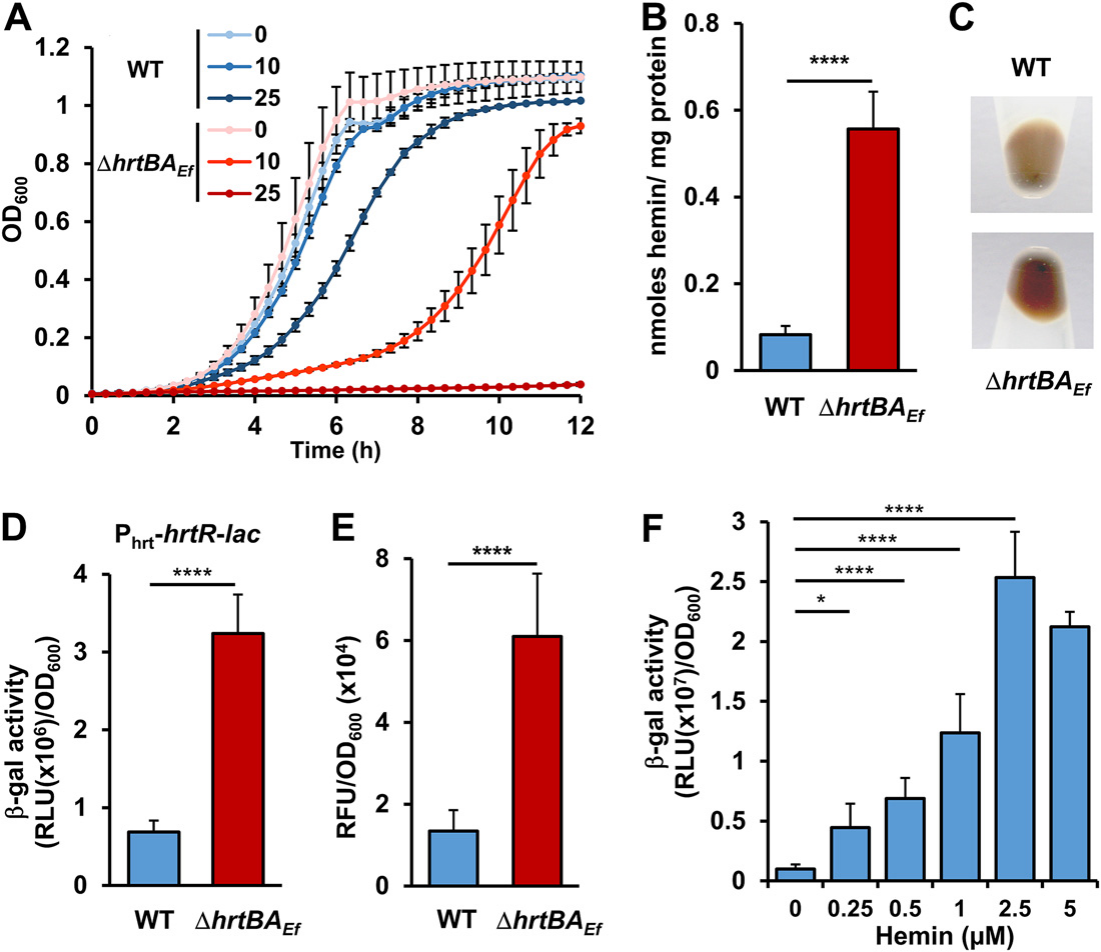
HrtBA*_Ef_* controls and responds to heme intracellular concentration. (A) Deletion of *hrtBA_Ef_* increases sensitivity to hemin toxicity. Overnight cultures of WT and Δ*hrtBA_Ef_* strains were diluted to an OD_600_ of 0.01 and grown with the indicated concentrations of hemin (in micromolar) for 10 h at 37°C in a microplate Spark spectrophotometer (Tecan). OD_600_ was measured every 20 min. Values are the means ± standard deviations (error bars) from three biological replicates. (B) Heme accumulates in the Δ*hrtBA_Ef_* strain. WT and Δ*hrtBA_Ef_* strains were grown to an OD_600_ of 0.5 prior to the addition of 5 μM hemin in the culture medium for an additional 1.5 h. Bacteria were pelleted by centrifugation, and heme content was determined by the pyridine hemochrome assay on cell lysates. Heme content was normalized to the protein concentration. Background from bacteria not incubated with hemin was subtracted. Results represent the means plus standard deviations (error bars) from three biological replicates. Statistical significance was determined by *t* test where **** = *P* < 0.0001. (C) Visualization of cellular heme accumulation in the Δ*hrtBA_Ef_* mutant. Cells, grown as described above for panel A, were incubated for 1.5 h with 5 μM hemin. The bacteria were photographed following centrifugation. The results are representative of three independent experiments. (D) HrtBA*_Ef_* reduces heme cytoplasmic concentration. WT and Δ*hrtBA_Ef_* strains carrying the intracellular sensor plasmid, pP_hrt_-*hrtR*-*lac* were grown as described above for panel B. β-Gal activity was quantified by luminescence in relative light units [RLU]) after 1.5 h of incubation with 5 μM hemin. Results represent the means plus standard deviations from three biological replicates. Statistical significance was determined by *t* test where **** = *P* < 0.0001. (E) HrtBA*_Ef_* prevents hemin-induced oxidative stress. WT and Δ*hrtBA_Ef_* strains were grown as described above for panel B with 5 μM hemin. Cells were washed with PBS plus 0.5% glucose, and ROS generation was quantified by the fluorescence of dihydrorhodamine 123. Results represent the means plus standard deviations from three biological replicates. Fluorescence background from bacteria not incubated with hemin was subtracted. Statistical significance was determined by *t* test where **** = *P* < 0.0001. (F) Induction of *hrtBA_Ef_* operon by hemin. The WT strain transformed with the reporter plasmid pP_hrtBA_-*lac* was grown, and β-gal activity was determined as described above for panel D following incubation with the indicated concentrations of hemin. Results represent the means ± standard deviations from three biological replicates. Statistical significance was determined by one-way analysis of variance (ANOVA) with Dunnett’s multiple-comparison test comparing each concentration of hemin to no-hemin control with statistical significance indicated as follows: *, *P* = 0.0202; ****, *P* < 0.0001.

E. faecalis OG1RF genome encodes two adjacent open reading frames (ORFs), OG1RF_RS02770 and OG1RF_RS02775 sharing, respectively 24% and 45% amino acid (AA) sequence identity with HrtB and HrtA from Staphylococcus aureus (18) (see Fig. S1A and S1B in the supplemental material). We thus verified the role of these ORFs, referred to as HrtB*_Ef_* and HrtA*_Ef_*, in heme efflux. Growth of an in-frame Δ*hrtBA_Ef_* deletion mutant was severely impaired at hemin concentrations ≥ 25 μM compared to the wild-type (WT) OG1RF strain (Fig. 1A). However, WT OG1RF could overcome up to 500 μM hemin, highlighting the involvement of HrtBA*_Ef_* in limiting heme toxicity in E. faecalis (Fig. S1C). The Δ*hrtBA_Ef_* mutant grown in 5 μM heme-containing medium accumulated about twofold more intracellular heme than the WT strain, as evaluated by the pyridine hemochrome assay (23) (Fig. 1B). This result correlated with the intense red color of culture pellets from the Δ*hrtBA_Ef_* mutant compared to the WT strain (Fig. 1C). Intracellular heme concentrations were also monitored using the intracytoplasmic heme sensor HrtR (19): β-galactosidase (β-gal) activity from the reporter plasmid P_hrt_-*hrtR*-*lac* was about 4 times higher in Δ*hrtBA_Ef_* compared to the WT exposed to 5 μM heme (Fig. 1D). Finally, accumulation of heme in the Δ*hrtBA_Ef_* mutant correlated with a more than twofold increase of cellular ROS generated by heme as shown by the fluorescence of dihydrorhodamine 123 (24) (Fig. 1E). In conclusion, E. faecalis expresses a functional HrtBA*_Ef_* heme efflux transporter that modulates intracellular heme levels, thus reducing oxidative stress.

10.1128/mBio.03392-20.4 FIG S1 HrtBA*_Ef_*, an S. aureus HrtBA-like efflux transporter. (A and B) Alignment of OG1RF_RS02770 and OG1RF_RS02775 with HrtB (A) and HrtA (B) from Staphylococcus aureus, respectively. AA sequences were aligned using Clustal W. Identical amino acids (*), conserved amino acids (:), and partially conserved amino acids (.) are indicated. (C) Growth of WT OG1RF strain with hemin. Overnight cultures of the WT strain were diluted to an OD_600_ of 0.01 and grown with the indicated concentrations of hemin (μM) for 15 h at 37°C in a microplate Spark spectrophotometer (Tecan). OD_600_ was measured every 20 min. Results represent the means ± standard deviations from three biological replicates. Download FIG S1, TIF file, 2.4 MB. Copyright © 2021 Saillant et al. 2021 Saillant et al. This content is distributed under the terms of the Creative Commons Attribution 4.0 International license.

Transcriptional regulation of *hrtBA_Ef_* by heme was then investigated using a *hrtBA_Ef_* promoter reporter, P_hrtBA_-*lac*. β-Gal expression in the WT strain was induced as a function of concentration between 0.1 and 2.5 μM hemin in the culture medium. Induction reached a maximum at concentrations below 5 μM (Fig. 1F). This concentration range is far below WT strain sensitivity to heme toxicity (≥25 μM) (Fig. 1A). We conclude that HrtBA*_Ef_* expression is induced at subtoxic heme concentrations.

### A new TetR regulator, FhtR, controls *hrtBA_Ef_* expression.

**FIG 2:**
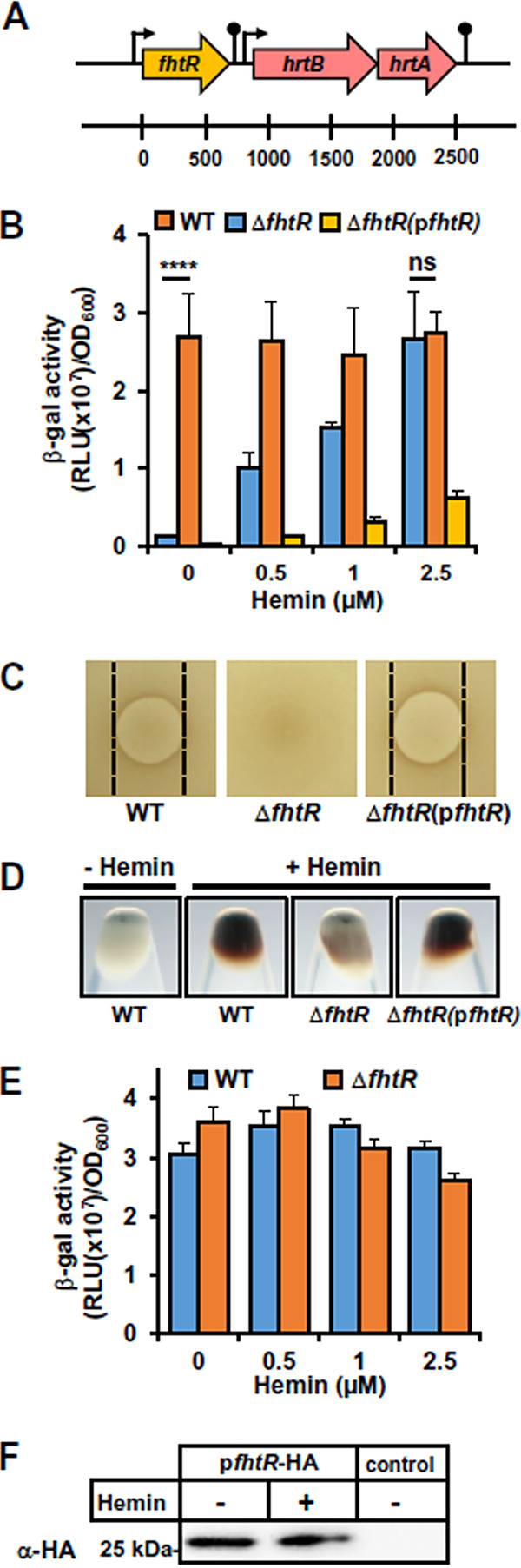
FhtR controls *hrtBA_Ef_* expression. (A) Schematic representation of the *fhtR* gene and *hrtBA_Ef_* operon. The *fhtR* gene (OG1RF_RS02765) encodes a TetR family transcriptional regulator. The *hrtB_Ef_* (OG1RF_RS02770) and *hrtA_Ef_* (OG1RF_RS02775) genes encode a permease and ATPase, respectively. (B) FhtR controls *hrtBA_Ef_* expression. WT and Δ*fhtR* strains carrying the reporter plasmid pP_hrtBA_-*lac* and a Δ*fhtR* strain carrying a plasmid, p*fhtR*, combining both P_hrtBA_-*lac* and P_fhtR_-*fhtR* were grown to an OD_600_ of 0.5, and β-gal expression was quantified by luminescence as reported in the legend to Fig. 1 with the indicated concentrations of hemin. Results represent the means plus standard deviations (error bars) from three biological replicates. Statistical significance was determined by *t* test as follows: ns, not significant (*P* > 0.5); ****, *P* < 0.0001. (C) *fhtR* deletion abrogates heme toxicity. Stationary-phase cultures of WT, Δ*fhtR*, and Δ*fhtR*(p*fhtR*) strains were plated on solid medium. Hemin (10 μl of a 1 mM stock solution) was pipetted directly onto plates, which were incubated for 24 h. Inhibition zones appear as a circular clearing in the center of each panel. No inhibition zone was observed for the Δ*fhtR* strain. The results are representative of three independent experiments. (D) Visualization of the impact of FhtR on heme accumulation. WT, Δ*fhtR*, and Δ*fhtR*(p*fhtR*) strains were grown and incubated with 5 μM hemin as described above for panel A. The bacteria were pelleted by centrifugation and photographed. The results are representative of three independent experiments. (E) FhtR expression is constitutively induced. β-Gal expression upon hemin addition to the culture medium in WT and Δ*fhtR* strains transformed with the pP_fhtR_-*lac* reporter plasmid was determined by luminescence as described in the legend to Fig. 1. Results represent the means plus standard deviations from three biological replicates. (F) *fhtR* transcription is not mediated by hemin. The Δ*fhtR* strain transformed with pP_fhtR_-*fhtR*-HA or carrying an empty vector (control) was used to monitor FhtR expression by Western blotting (WB) using an antihemagglutinin (anti-HA) antibody (α-HA). Bacteria were grown to an OD_600_ of 0.5 and incubated with 2.5 μM hemin for 1.5 h. Sodium dodecyl sulfate-polyacrylamide gel electrophoresis (SDS-PAGE) was performed on cell lysates (80 μg per lane). The results are representative of three independent experiments.

The above findings prompted us to investigate the mechanism of *hrtBA_Ef_* induction. Several Gram-positive pathogens regulate *hrtBA_Ef_* via an adjacent two-component system HssR and HssS (14, 20, 21). No *hssR hssS* genes were identified in or near the *hrtBA_Ef_* operon in E. faecalis OG1RF or other E. faecalis genomes. However, a monocistronic gene encoding a TetR family transcriptional regulator, OG1RF_RS02765, is adjacent to *hrtBA_Ef_* (Fig. 2A), sharing no significant AA identity with the *hrtBA* regulator, HrtR, in Lactococcus lactis (19). We hypothesized that OG1RF_RS02765 was the transcriptional regulator of *hrtBA_Ef_* and tentatively named it FhtR (for faecalis heme transport regulator) (Fig. 2A).

To investigate the role of FhtR in heme-dependent transcription of *hrtBA_Ef_*, an *fhtR* in-frame deletion in strain OG1RF (Δ*fhtR*) was constructed and transformed either with pP_hrtBA_-*lac* or p*fhtR* encompassing both (pP_hrtBA_-*lac* and P_fhtR_*-fhtR*) expression cassettes (Fig. 2B). In contrast to the WT strain, β-gal was expressed independently of heme in Δ*fhtR*(pP_hrtBA_-*lac*) (Fig. 2B). Transformation of p*fhtR* with Δ*fhtR* led to overcomplementation compared to the WT(pP_hrtBA_-*lac*) strain (Fig. 2B). Moreover, on solid medium, Δ*fhtR* exhibited a complete absence of sensitivity to heme compared to the WT and complemented Δ*fhtR*(p*fhtR*) strains (Fig. 2C). Similar results were obtained in liquid culture (Fig. S2). These results are in line with the observation that heme accumulation is reduced in the Δ*fhtR* mutant strain compared to the WT or Δ*fhtR*(p*fhtR*) strain (Fig. 2D) and that HrtBA*_Ef_* may be constitutively expressed in the Δ*fhtR* mutant (Fig. 2B). Indeed, P_fhtR_ was constitutively transcribed, with no effects of heme, nor of FhtR expression as shown using P_fhtR_-*lac* as the reporter (Fig. 2E), and by Western blotting (WB) using FhtR-hemagglutinin (HA) tagged fusion expressed from P_fhtR_ (Fig. 2F). We conclude that E. faecalis uses a constitutively expressed, unique intracellular heme sensor, FhtR, to control *hrtBA_Ef_* expression.

10.1128/mBio.03392-20.5 FIG S2 Planktonic growth of WT, Δ*fhtR*, and Δ*fhtR*(p*fhtR*) strains with hemin. Overnight cultures of WT, Δ*fhtR*, and Δ*fhtR*(p*fhtR*) strains were diluted to an OD_600_ of 0.01 and grown with or without 200 μM hemin for 15 h at 37°C in a microplate Spark spectrophotometer (Tecan). OD_600_ was measured every 20 min. Results represent the means ± standard deviations from three biological replicates. Download FIG S2, TIF file, 2.2 MB. Copyright © 2021 Saillant et al. 2021 Saillant et al. This content is distributed under the terms of the Creative Commons Attribution 4.0 International license.

### FhtR is a heme binding protein.

**FIG 3:**
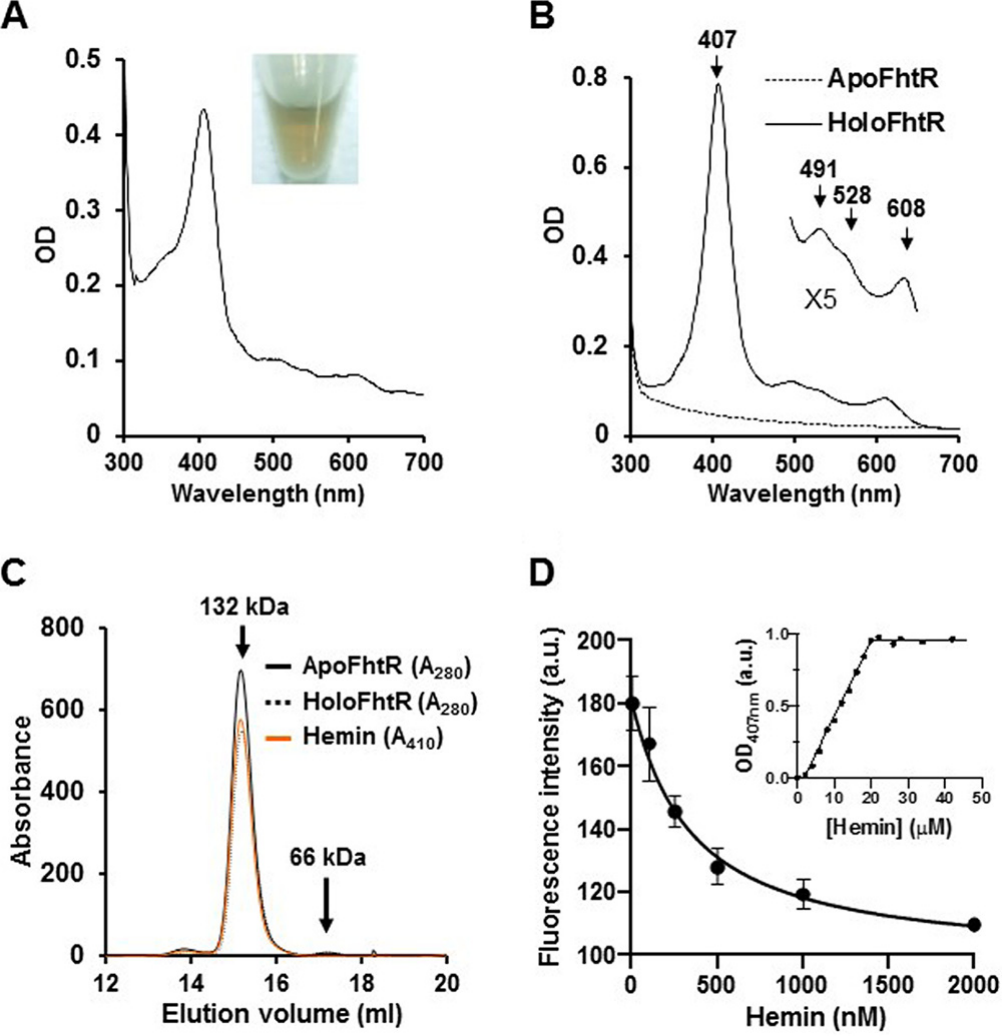
FhtR binds heme. (A) UV-visible absorption spectra of MBP-FhtR as purified from E. coli. UV-visible spectra of 30 μM (in 200 μl) MBP-FhtR was obtained in a microplate spectrophotometer (Spark; Tecan) and normalized to an OD_280_ of 1. (Inset) Photograph of the purified MBP-FhtR. Results are representative of three independent experiments. (B) UV-visible spectra of apoMBP-FhtR complexed with hemin. MBP-FhtR was purified from E. coli (*hemA*::*kan*) strain as an apoprotein (dashed line) that was mixed with equimolar concentration of hemin. Spectra was obtained as described above for panel A with 20 μM complex and normalized to an OD_280_ of 1. (Inset) Magnification of the 500- to 700-nm region. Results are representative of three independent experiments. (C) Size-exclusion chromatography of apo- and holo-MBP-FhtR. MBP-FhtR was purified and complexed with hemin as described above for panel B. 40 μM of the complex in 100 μl was loaded on a Superdex 200 Increase 10/300 GL gel filtration column (GE Healthcare) in 20 mM HEPES (pH 7), 300 mM NaCl buffer. Protein and heme content were analyzed at OD_280_ and OD_410_. The results are representative of three independent experiments. (D) Titration of MBP-FhtR with hemin followed by fluorescence and absorbance (inset). For the fluorescence experiment, 50 nM ApoMBP-FhtR purified from E. coli (*hemA*::*kan*) as described for panel B were titrated with increasing increments of hemin. Fluorescence intensity (in arbitrary units [a.u.]) was recorded and plotted against hemin concentration. The experiment was repeated three times, fitted using the nonlinear regression function of GraphPad Prism 4 software, and gave a *K_d_* of 310 nM. The inset depicts the absorbance at 407 nm of ApoMBP-FhtR plotted against hemin concentration. The curve is representative of 10 independent experiments and was fitted using the nonlinear regression function of GraphPad Prism 4 software, which determined that the stoichiometry of the FhtR-hemin complex was 1:1.

Members of the TetR family of transcriptional regulators act as chemical sensors (25, 26). Ligand binding alleviates TetR protein interactions with their respective operators, leading to promoter induction (25, 26). To verify that heme was the signal that relieves FhtR-mediated *hrtBA_Ef_* repression, recombinant FhtR was purified as a fusion to the maltose binding protein (MBP-FhtR) from Escherichia coli. MBP-FhtR appeared green (Fig. 3A, inset), and its UV-visible spectrum exhibited a strong Soret band, suggesting that FhtR scavenges endogenously produced heme (Fig. 3A). To purify an apoFhtR, MBP-FhtR was expressed from a heme synthesis-deficient E. coli strain (*hemA*::*kan*) (Fig. 3B, dashed line). The purified protein bound hemin *b in vitro* (i.e., noncovalently) with a similar UV-visible spectrum as observed above for *in vivo*-bound heme: a Soret band at 407 nm and Q bands at 491 nm, 528 nm, and 608 nm (Fig. 3B, holoFhtR). Size-exclusion chromatography profiles showed that both apo- and holo-MBP-FhtR eluted as a single peak corresponding to the size of a dimer (132 kDa), in line with other TetR regulators (Fig. 3C) (25). The 608-nm charge transfer band and Soret at 407 nm are indicative of a ferric high-spin tyrosinate-ligated heme where heme is anchored through a proximal tyrosinate side chain (27, 28). Hemin pentacoordinate high*-*spin ligation to FhtR was further confirmed by electron paramagnetic resonance (EPR) spectroscopy (see below). The heme dissociation coefficient (*K_d_*) was 310 nM as determined by MBP-FhtR fluorescence quenching over increasing concentrations of hemin (Fig. 3D). Heme titration by differential absorption spectroscopy at 407 nm showed that the saturation point corresponded to the binding of one molecule of hemin per MBP-FhtR monomer (Fig. 3D, inset). Altogether, these data demonstrate that FhtR is a heme binding protein, suggesting that heme interaction is the primary event leading to activation of *hrtBA_Ef_* transcription.

### Tyrosine 132 is a crucial heme axial ligand in FhtR.

**FIG 4:**
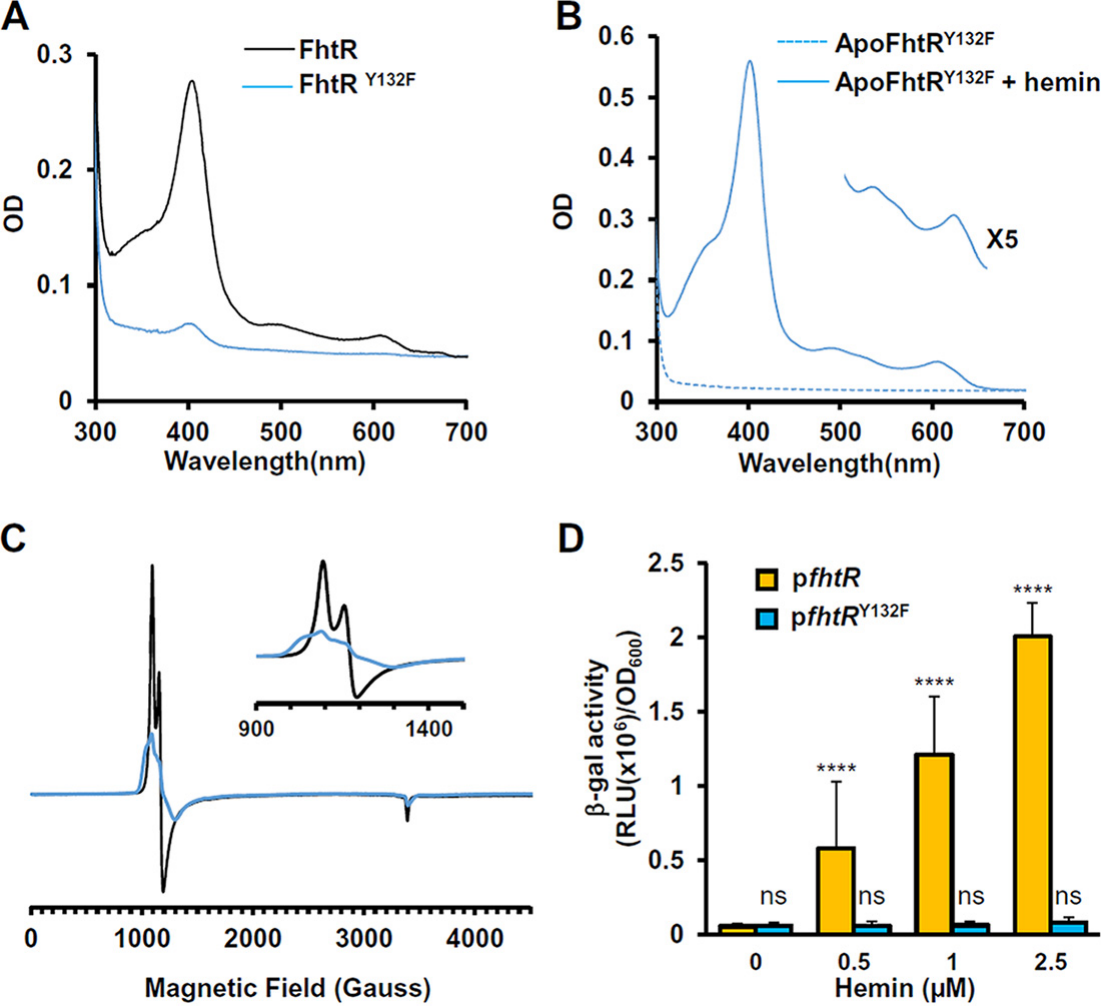
Ligation of FhtR with hemin implicates the tyrosine Y132. (A) Comparative UV-visible absorption spectra of 20 μM MBP-FhtR and MBP-FhtR^Y132F^ purified from E. coli. UV-visible spectra were performed as described in the legend to Fig. 3A. The results are representative of three independent experiments. (B) UV-visible spectra of apoMBP-FhtR^Y132F^ complexed with hemin. MBP-FhtR^Y132F^ was purified from E. coli (*hemA*::*kan*) strain as an apoprotein (dashed line) that was mixed with equimolar concentration of hemin (holoMBP-FhtR). Spectra were obtained as described for panel A with 20 μM complex and normalized to an OD_280_ of 1. (Inset) Magnification of the 500- to 700-nm region. Results are representative of three independent experiments. (C) EPR spectroscopy of MBP-FhtR and MBP-FhtR^Y132F^ in complex with hemin. EPR spectra of 60 μM bound hemin to WT (black line) and Y132 mutant (blue line) MBP-FhtR in 20 mM HEPES (pH 7) and 300 mM NaCl were recorded. (Inset) Magnification of the 900- to 1,500-gauss magnetic field range. The results are representative of three independent experiments. (D) Induction of the *hrtBA_Ef_* operon by hemin. β-gal activity from the Δ*fhtR* strain transformed either with pP_hrtBA_-*lac*, P_fhtR_-*fhtR* or pP_hrtBA_-*lac*, or P_fhtR_-fhtR^Y132F^ was determined as described in the legend to Fig. 1C following incubation with the indicated concentrations of hemin. Results represent the means plus standard deviations from three biological replicates. Statistical significance was determined by one-way analysis of variance (ANOVA) with Dunnett’s multiple-comparison test comparing each concentration of hemin to p*fhtR* (0 μM) control with statistical significance indicated as follows: ns, not significant (*P* > 0.5); ****, *P* < 0.0001.

According to UV-visible and EPR spectra, the likely candidate for axial ligand of oxidized heme is a tyrosine (Y) (see above). Several Y residues present in FhtR were substituted to phenylalanine (F) (Fig. S3A, in blue). F and Y both have phenyl ring structures, so that F substitution minimizes an impact on FhtR conformation. Although F lacks the hydroxyl group that coordinates heme, FhtR heme binding was not modified for several mutants tested individually (Fig. S3A). Only FhtR^Y132F^ was purified from E. coli with a strong decrease in heme content compared to WT MBP-FhtR, indicating a loss of heme affinity *in vivo* (Fig. 4A). Surprisingly, apoMBP-FhtR^Y132F^ purified from *hemA*::*kanA* E. coli exhibited similar UV-visible spectra (Fig. 4B) and *K_d_* upon hemin addition (data not shown), questioning the implication of this tyrosine in heme binding. The role of Y132 was further analyzed by EPR spectroscopy (Fig. 4C). HoloMBP-FhtR exhibited an axial high-spin (*S *=* *5/2) heme signal with two well-defined resonances at around *g* ∼ 6 (with a crossing point at 1,190 G) (Fig. 4C, inset) and a resonance at *g* ∼ 2 (∼3,390 G), indicative of a 5-coordinated Fe^III^ structure. Although the UV-visible spectra of FhtR and FhtR^Y132F^ supplemented with hemin do not differ to a detectable level, the EPR spectra of FhtR^Y132F^ was significantly modified, thus showing that either the ligand of the iron has been exchanged for another one or more likely, the interaction of the axial ligand with its environment has been modified. To conciliate these results, it is possible that while Y132 is the primary ligand, another distal ligand can take over ligation in the Y132F mutant to become the dominant ligand *in vitro* (meanwhile hydrophobic contacts would ensure retention of the binding affinity).

10.1128/mBio.03392-20.6 FIG S3 Hemin binding to FhtR implicates the tyrosine Y132. (A) E. faecalis OG1RF FhtR amino acid sequence. Predicted nucleotide (Nt) binding region of the TetR family OG1RF_RS02765 ORF to its target DNA is shown in orange. Tyrosines are shown in blue, and Y132 is shown in bold type. (B) FhtR^Y132F^ is expressed similarly to WT FhtR. The Δ*fhtR* mutant was complemented either with p*fhtR-*HA or p*fhtR*^Y132F^-HA plasmid. FhtR and FhtR^Y132F^ expression were monitored by WB with an anti-HA antibody. Bacteria were grown to an OD_600_ of 0.5 and incubated with 2.5 μM hemin for 1.5 h. SDS-PAGE was performed on cell lysates (35 μg protein) per lane. The results are representative of three independent experiments. Download FIG S3, TIF file, 2.3 MB. Copyright © 2021 Saillant et al. 2021 Saillant et al. This content is distributed under the terms of the Creative Commons Attribution 4.0 International license.

We then compared FhtR and FhtR^Y132F^ activities *in vivo*. The Δ*fhtR* mutant was complemented either with p*fhtR* (pP_hrtBA_-*lac*; P_fhtR_-*fhtR*) or p*fhtR*^Y132F^ (pP_hrtBA_-*lac*; P_fhtR_-*fhtR*^Y132F^), and β-gal expression was monitored upon hemin addition to medium (Fig. 4D). WT FhtR and FhtR^Y132F^ were expressed to similar levels as confirmed on WB (Fig. S3B). Expression of both proteins prevented *hrtBA_Ef_* transcription in the absence of heme, in contrast to full expression in Δ*fhtR* (Fig. 4D). WT FhtR and FhtR^Y132F^ were expressed to similar levels as confirmed on WB (Fig. S3B). However, hemin addition led to P_hrtBA_-*lac* expression in the strain carrying WT FhtR, but not FhtR^Y132F^, suggesting that heme derepression was impaired (Fig. 4D). Altogether, these data specify FhtR Y132 as a critical residue in the coordination of heme with FhtR, which enables *hrtBA_Ef_* transcription.

### FhtR controls *hrtBA_Ef_* transcription by binding two distinct 14-nt palindromic repeat sequences.

**FIG 5:**
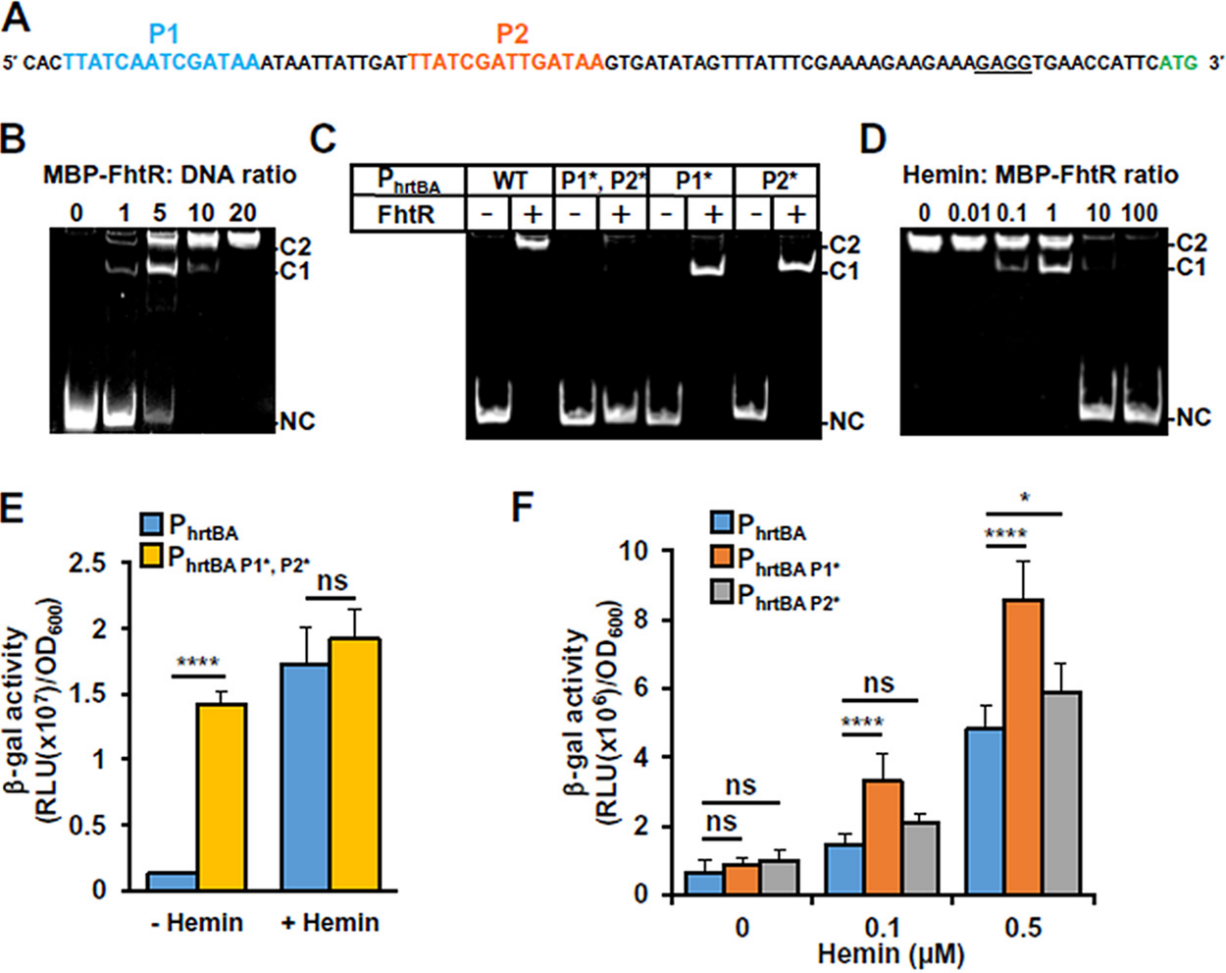
FhtR controls *hrtBA_Ef_* transcription via binding to two repeated 14-nt palindromic sequences. (A) Two 14-nt palindromic motifs are present upstream of *hrtBA_Ef_*. The two palindromes are shown in blue (P1) and in red (P2); the ribosome binding sequence (RBS) is underlined, and the start codon is shown in green. (B) FhtR binds to the promoter region of *hrtBA_Ef_*. EMSA shows binding of FhtR to P_hrtBA_. The *hrtBA_Ef_* promoter fragment (0.25 pmol) was incubated with increasing amounts of MBP-FhtR as indicated in molar ratios. DNA shift was visualized with GelRed (Biotium) following PAGE. The two shifted DNA-protein complexes (C1 and C2) and noncomplexed DNA (NC) are indicated. The results are representative of at least three independent experiments. (C) Roles of P1 and P2 on FhtR binding to the promoter region of *hrtBA_Ef_*. EMSA was performed as described for panel B with either the native P_hrtBA_ DNA fragment (WT) (as in panel A) or mutated fragments P_hrtBA P1*, P2*_, P_hrtBA P1*_, and P_hrtBA P2*_. MBP-FhtR: DNA (2.5 pmol: 0.25 pmol). The results are representative of at least three independent experiments. (D) Effect of hemin on the binding of FhtR to the *hrtBA_Ef_* promoter. The *hrtBA_Ef_* promoter DNA (0.25 pmol) was incubated with 2.5 pmol of MBP-FhtR together with increasing amounts of hemin as indicated (molar ratios) and analyzed by EMSA as described in the legend to panel B. The results are representative of at least three independent experiments. (E) Substitution of the two palindromic nucleotide sequences, P1 and P2, in P_hrtBA_ abrogates FhtR-mediated control of *hrtBA_Ef_* transcription. The WT strain was transformed either with the reporter plasmid pP_hrtBA_-*lac* or pP_hrtBA P1*P2*_ *-lac*. β-Gal activity was determined as described in the legend to Fig. 1 following incubation with 2.5 μM hemin. Results represent the means plus standard deviations from three biological replicates. Statistical significance was determined by *t* test with statistical significance indicated as follows: ns, not significant (*P* > 0.5); ****, *P* < 0.0001. (F) Substitution of either P1 or P2 nucleotide sequences in P_hrtBA_ enhances its transcriptional activation by hemin. The WT strain was transformed either with the reporter plasmid pP_hrtBA_-*lac*, pP_hrtBA P1*_ *-lac*, or pP_hrtBA P2*_ *-lac*. β-Gal activity was determined as described in the legend to Fig. 1 following incubation with hemin. Results represent the means plus standard deviations from three biological replicates. Statistical significance was determined by one-way ANOVA with Tukey’s multiple-comparison test with significance indicated as follows: ns, not significant (*P* > 0.5); *, *P* = 0.0140; ****, *P* < 0.0001.

TetR family operators usually comprise a 10- to 30-nucleotide (nt) inverted repeat sequence with internal palindromic symmetry (25). Two such 14-nt-long palindromes were identified in the −10/−35 region of the *hrtBA_Ef_* promoter (called P1 and P2; Fig. 5A). An electrophoretic mobility shift assay (EMSA) was performed with apoMBP-FhtR, using a 325-bp DNA segment comprising the *hrtBA_Ef_* promoter (Fig. 5B) or a segment covering the internal *hrtB* region as a control (Fig. S4). FhtR-specific interaction with the P_hrtBA_ DNA segment confirmed FhtR binding specificity. The shifted DNA migrated as two distinct bands (C1 and C2), in proportions that depended on the MBP-FhtR: DNA ratio (Fig. 5B), plausibly revealing that FhtR complexes with either one or two palindromes (Fig. 5B). To test this, we performed random substitutions of P1 and/or P2 nucleotides (P1* and P2*) and analyzed DNA shifts by EMSA. Replacement of both distal and proximal operators (P_hrtBA P1*, P2*_) abolished the FhtR-induced DNA shift, confirming the role of palindromes in the interaction of FhtR with P_hrtBA_ (Fig. 5C). Single replacement of P1 (P_hrtBA P1*_) or P2 (P_hrtBA P2*_) resulted in complete DNA shifts that migrated faster in the gel (C1) than seen with the native nucleotide sequence (Fig. 5C). We conclude that both P1 and P2 are FhtR binding sites.

10.1128/mBio.03392-20.7 FIG S4 MBP-FhtR interaction with P_hrtBA_ is specific. The *hrtBA_Ef_* promoter fragment (PF_hrtBA_) (0.25 pmol) or a similar size nucleotide (nt) sequence in the coding region of *hrtB_Ef_* (CR_hrtB_) were incubated with 2.5 pmol of MBP-FhtR. Both DNA fragments were PCR amplified from OG1RF genomic DNA with the oligonucleotides (O21-O22) and (O23-O24), respectively. EMSA was performed as described in the legend to Fig. 4, and DNA shift was visualized with GelRed (Biotium) following PAGE. The shifted DNA-protein complex (C2) and the noncomplexed DNA (NC) are indicated. The results are representative of three independent experiments. Download FIG S4, TIF file, 1.7 MB. Copyright © 2021 Saillant et al. 2021 Saillant et al. This content is distributed under the terms of the Creative Commons Attribution 4.0 International license.

We then tested the effects of heme on FhtR binding by EMSA. Addition of hemin to MBP-FhtR abolished the formation of the DNA-FhtR complex, as seen by the progressive disappearance of band shifts with increasing hemin concentrations (Fig. 5D). Complete release of FhtR from P_hrtBA_ was obtained when hemin was in 10-fold molar excess over FhtR (Fig. 5D). Both C1 and C2 complexes were revealed when intermediate amounts of heme were added (0.1 and 1 μM; Fig. 5D). This suggests the release of MBP-FhtR from only one operator depending on the saturation level of FhtR with hemin.

The role of the two operators in the control of *hrtBA_Ef_* was investigated *in vivo*, using P_hrtBA_ or a P_hrtBA P1*, P2*_ promoter variant to control *lac* gene (Fig. 5E). In contrast to pP_hrtBA_-*lac*, which was strongly induced with 1 μM hemin, P_hrtBA P1*, P2*_-*lac* was constitutively expressed (Fig. 5E). Finally, the role of each operator was investigated (Fig. 5F). In the absence of heme, either P1 or P2 is sufficient for full P_hrtBA_ repression by FhtR. Release of the promoter was facilitated in the presence of only P1 or P2 as shown with increased transcriptional activities of P_hrtBA P1*_ or P_hrtBA P2*_ in the presence of hemin compared to native P_hrtBA_ (Fig. 5F). We propose that the presence of two operators provides strong repression of the *hrtBA_Ef_* promoter, thus preventing transcriptional leakage and allowing for fine tuning of HrtBA*_Ef_* expression. Taken together, these results demonstrate that FhtR is a heme sensor that directly controls heme homeostasis by regulating *hrtBA_Ef_* transcription.

### FhtR controls HrtBA*_Ef_*, the gatekeeper of intracellular heme availability.

**FIG 6:**
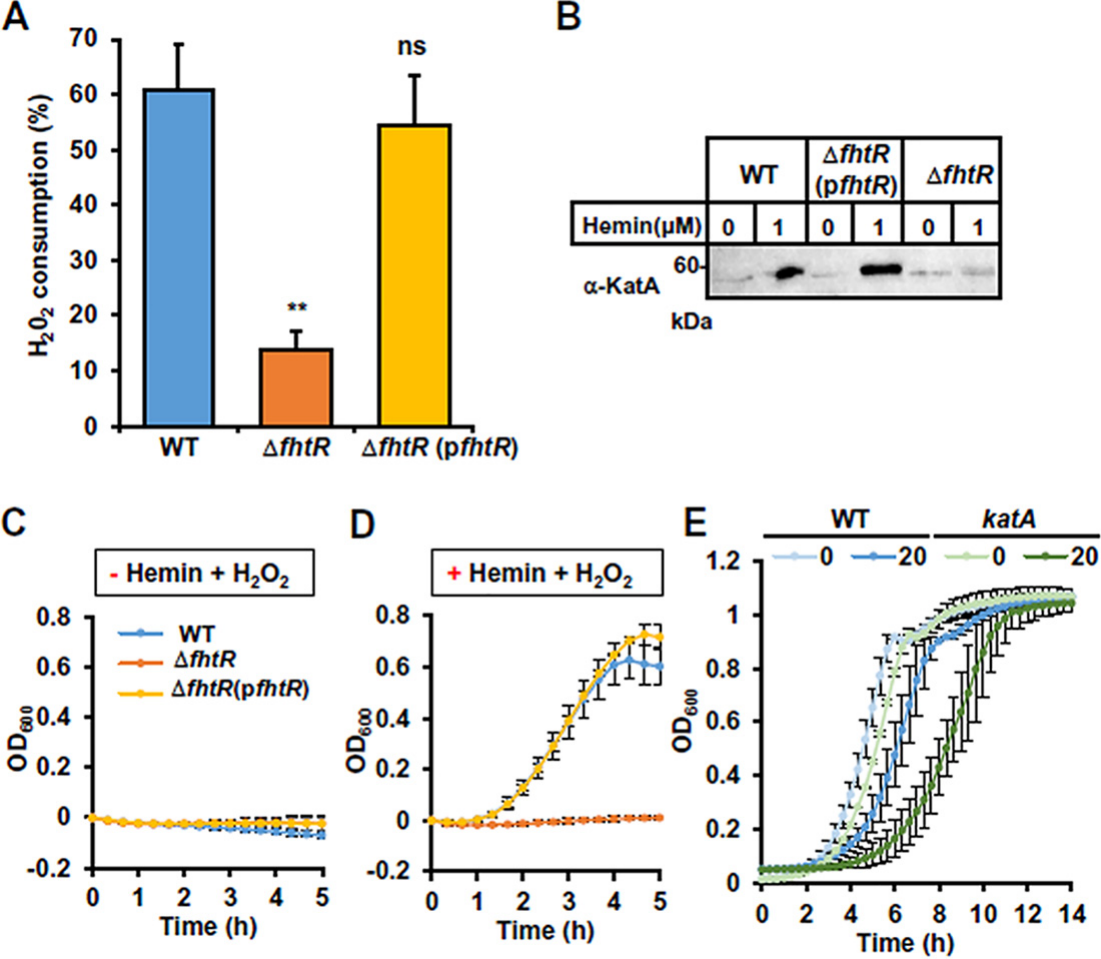
FhtR controls intracellular utilization of heme. (A) *fhtR* deletion limits heme-dependent KatA activity. KatA enzymatic activity in E. faecalis was assessed on WT, Δ*fhtR*, and Δ*fhtR*(p*fhtR*) grown overnight (ON) with 1 μM hemin. Catalase activity was determined on an equivalent number of bacteria incubated with 100 μM H_2_O_2_ for 1 h with the spectrophotometric FOX1 method based on ferrous oxidation in xylenol orange. Results are expressed as the percentage of H_2_O_2_ metabolized in respective strains grown without hemin. Results represent the means plus standard deviations from three biological replicates. Statistical significance was determined by one-way analysis of variance (ANOVA) with Dunnett’s multiple-comparison test comparing each strain to the WT strain control with significance indicated as follows: ns, *P* > 0.05; **, *P* = 0.008. (B) Expression of KatA is impaired in the Δ*fhtR* mutant. Equivalent amounts of protein (20 μg) from lysates of WT, Δ*fhtR*, and Δ*fhtR*(p*fhtR*) strains as described for panel A were separated by SDS-PAGE, and immunoblots were probed with an anti-KatA polyclonal antibody. The presented results are representative of three independent experiments. (C and D) The Δ*fhtR* mutant is hypersensitive to H_2_O_2_. ON cultures of WT, Δ*fhtR*, and Δ*fhtR*(p*fhtR*) strains were diluted to an OD_600_ of 0.01 and grown to an OD_600_ of 0.5 in the absence of hemin (C) or in the presence of 1 μM hemin (D). Cultures were distributed in wells on a 96-well plate and supplemented with 2.5 mM H_2_O_2_ or not supplemented with H_2_O_2_. OD_600_ was monitored every 20 min in a microplate spectrophotometer (Spark; Tecan). OD_600_ at time zero was normalized to 0. Results represent the means ± standard deviations from three biological replicates. (E) KatA limits heme toxicity. ON cultures of WT and *katA*::*tetR* strains were diluted to an OD_600_ of 0.01 and grown in 96-well plate in the presence of the indicated concentration of hemin as described for panel D. Results represent the means ± standard deviations from three biological replicates.

Our observation that FhtR regulates intracellular heme pools even at low heme concentrations led us to hypothesize that FhtR controls intracellular heme availability in E. faecalis. We tested this possible role of FhtR on the E. faecalis endogenous heme-dependent catalase (KatA). While *katA* transcription is not susceptible to heme induction, KatA protein stability relies on the presence of heme (10, 12). KatA-mediated H_2_O_2_ catalysis was measured in WT, Δ*fhtR*, and Δ*fhtR*(p*fhtR*) strains (Fig. 6A). In the absence of hemin, H_2_O_2_ consumption was at a basal level (Fig. S5A), thus excluding major contributions of other enzymes in our conditions. In the presence of 1 μM hemin (Fig. 6A), the Δ*fhtR* mutant exhibited about 30% catalase activity compared to WT and complemented Δ*fhtR*(p*fhtR*) strains, as evaluated by the percentage of catabolized H_2_O_2_ (Fig. 6A). This was further confirmed by comparing the amounts of KatA (holoKatA) by WB, using anti-KatA antibody (kindly provided by L. Hederstedt). In the absence of hemin, KatA was expressed at low levels in WT, Δ*fhtR*, and Δ*fhtR*(p*fhtR*) strains (Fig. 6B). Comparatively, addition of hemin strongly increased the amounts of KatA in WT and complemented Δ*fhtR*(p*fhtR*) strains, but not in the Δ*fhtR* mutant (Fig. 6B). Low KatA availability in the Δ*fhtR* mutant is readily explained by constitutive heme efflux (via HrtBA*_Ef_*), and consequently depleted intracellular heme pools in this mutant. We then evaluated the survival capacity of E. faecalis OG1RF WT, Δ*fhtR*, and Δ*fhtR*(p*fhtR*) strains when challenged with 2.5 mM H_2_O_2_. In the absence of hemin, all strains grew equivalently without H_2_O_2_ (Fig. S5B). In contrast, while hemin addition rescued the survival of both the WT and Δ*fhtR*(p*fhtR*) strains, the Δ*fhtR* strain remained hypersensitive to H_2_O_2_ (Fig. 6C and D). Deletion of *hrtBA_Ef_* in the Δ*fhtR* strain (Δ*fhtR* Δ*hrtBA_Ef_*) restored the survival capacity in the presence of hemin (Fig. S5C). Thus, poor survival of Δ*fhtR* reflects the lack of heme needed to stabilize KatA (Fig. 6D). Finally, the OG1RF mutant (*katA*::*tetR*) was hypersensitive to hemin toxicity, showing that KatA was required for controlling oxidative stress generated by heme (Fig. 1E and Fig. 4E). Taken together, these results identify FhtR as the direct and indirect regulator of HrtBA*_Ef_*-mediated heme efflux and KatA activity, respectively, with both mechanisms lowering heme stress in E. faecalis OG1RF. FhtR is thus a key mediator of heme homeostasis, and consequently, of oxidative stress response in E. faecalis generated by H_2_O_2_.

10.1128/mBio.03392-20.8 FIG S5 Control experiments for the study of KatA and FhtR interplay. (A) H_2_O_2_ background consumption in WT, Δ*fhtR*, and Δ*fhtR*(p*fhtR*) strains grown in the absence of hemin. Catalase activity was determined on equivalent number of bacteria from ON cultures incubated with 100 μM H_2_O_2_ for 30 min with the spectrophotometric FOX1 method based on ferrous oxidation in xylenol orange as described in the legend to Fig. 6. Results expressed as the percentage of H_2_O_2_ concentration metabolized in respective strains. Results represent the means ± standard deviations from three biological replicates. Statistical significance was determined by one-way analysis of variance (ANOVA) with Dunnett’s multiple-comparison test comparing each strains to the WS strain control with significance indicated as follows: ns, not significant (*P* > 0.05). (B) Control growth curves of WT, Δ*fhtR*, and Δ*fhtR*(p*fhtR*) strains. Overnight cultures were diluted to an OD_600_ of 0.01 in M17G and grown to an OD_600_ of 0.5. Cultures were distributed in a 96-well plate and incubated at 37°C in a microplate Spark spectrophotometer (Tecan). OD_600_ was measured every 20 min. OD_600_ at time 0 was normalized to 0. Results represent the means ± standard deviations from three biological replicates. (C) In the absence of HrtBA*_Ef_* expression, FhtR has no impact on KatA. Δ*hrtBA_Ef_* and Δ*fhtR*Δ*hrtBA_Ef_* strains were grown as described for panel B with 1 μM hemin added at an OD_600_ of 0.01. OD_600_ at time 0 was normalized to 0. Data are the means ± standard deviations from biological triplicates. Download FIG S5, TIF file, 2.9 MB. Copyright © 2021 Saillant et al. 2021 Saillant et al. This content is distributed under the terms of the Creative Commons Attribution 4.0 International license.

### Heme sensing in the gastrointestinal tract.

**FIG 7:**
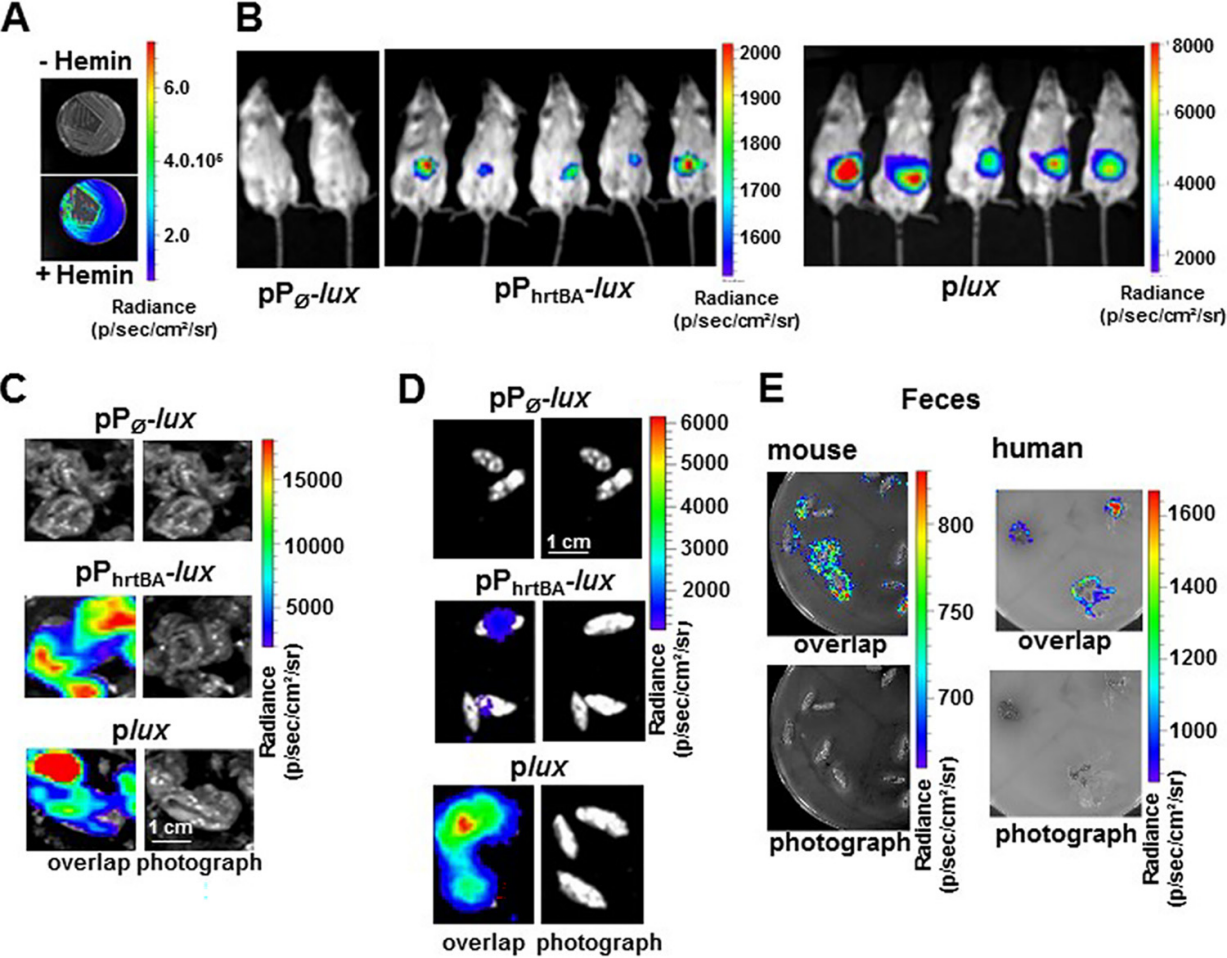
HrtBA*_Ef_* is induced in the gastrointestinal environment. (A) Heme-dependent light emission by the heme sensing WT(pP_hrtBA_-*lux*) strain. WT(pP_hrtBA_-*lux*) was plated on M17G agar plates in the presence (+) or absence (−) of 20 μM hemin. Plates were incubated at 37°C for 24 h, and luminescence was visualized using an IVIS 200 luminescence imaging system (acquisition time, 1 min; binning 8). Radiance is shown in photons per second per square centimeter per steradian. The results are representative of three independent experiments. (B) Heme sensing by E. faecalis over the course of intestinal transit. Female BALB/c mice were force fed with 10^8^ CFU of WT(pP_Ø_-*lux*) (control strain), WT(pP_hrtBA_-*lux*) (sensor strain), or WT(p*lux*) (tracking strain). At 6 h postinoculation, anesthetized mice were imaged in the IVIS 200 system (acquisition time, 20 min; binning 16). The figure shows representative animals corresponding to a total of 15 animals for each condition in three independent experiments. (C) Ceca exhibit high heme sensing signal. Animals as described above for panel B were euthanized and immediately dissected. Isolated GITs were imaged as described above for panel B. Ceca that exhibited most of the luminescence are shown (acquisition time, 5 min; binning 8). Bar = 1 cm. (D) Visualization of heme sensing in feces collected from mice following ingestion of WT(pP_Ø_-*lux*), WT(pP_hrtBA_-*lux*), or WT(p*lux*). WT(pP_hrtBA_-*lux*) as described above for panel B were collected 6 to 9 h after gavage. Feces were imaged as described above for panel B (acquisition time, 20 min; binning 16). Bar = 1 cm. Results are representative of three independent experiments. (E) Human and mouse fecal samples activate heme sensing. Human feces from three healthy human laboratory volunteers and mouse feces from 6-month-old female BALB/c mice were deposited on M17G agar plates layered with soft agar containing WT OG1RF (pP_hrtBA_-*lux*). Plates were incubated at 37°C for 16 h and imaged in the IVIS 200 system (acquisition time, 10 min; binning 8). The figure shows representative results of a total of three independent experiments.

E. faecalis is a normal resident of the GIT of vertebrates, an ecosystem where heme is available (29–32). We therefore investigated whether *hrtBA_Ef_*-mediated heme management is required by E. faecalis in the GIT in a murine gastrointestinal model. We generated E. faecalis OG1RF strains expressing the *luxABCDE* (*lux*) operon from Photorhabdus luminescens driven by the following: (i) P_hrtBA_ (pP_hrtBA_-*lux*), which emits light specifically in the presence of hemin (Fig. 7A); (ii) a constitutive promoter P23 (p*lux*), constitutively emitting light for bacterial tracking (14); or (iii) a control promoterless vector, pP_∅_-*lux*. Cultures of these strains were orally inoculated in the digestive tracts of mice, and light emission from whole live animals was measured in an *in vivo* imaging system IVIS200, 6 h postinoculation (Fig. 7B). This time delay corresponded to the maximum light emission from the tracking strain OG1RF(p*lux*) (Fig. S6A). Luminescence signaling from the ingested E. faecalis pP_hrtBA_-*lux* heme sensor strain also localized in the abdomen, similar to the tracking strain (Fig. 7B). Examination of dissected organs revealed that the heme sensor-associated luminescence was mainly detected in the cecum (Fig. 7C), correlating with the high bacterial load of this organ [WT(p*lux*); Fig. 7C]. A significant signal was also detected in the feces from inoculated animals, further highlighting that E. faecalis was able to scavenge and internalize heme within the digestive tract to induce *hrtBA_Ef_* expression (Fig. 7D). Finally, mice and human fecal samples (as well as fecal waters [Fig. S6B and S6C]) from healthy individuals were able to induce luminescence from WT(pP_hrtBA_-*lux*) *in vitro*, excluding the possibility that induction of P_hrtBA_ *in vivo* could result from the inoculation procedure (Fig. 7E). Therefore, FhtR heme sensor activity is active and relevant to E. faecalis heme management in the lumen of the GIT.

**FIG 8:**
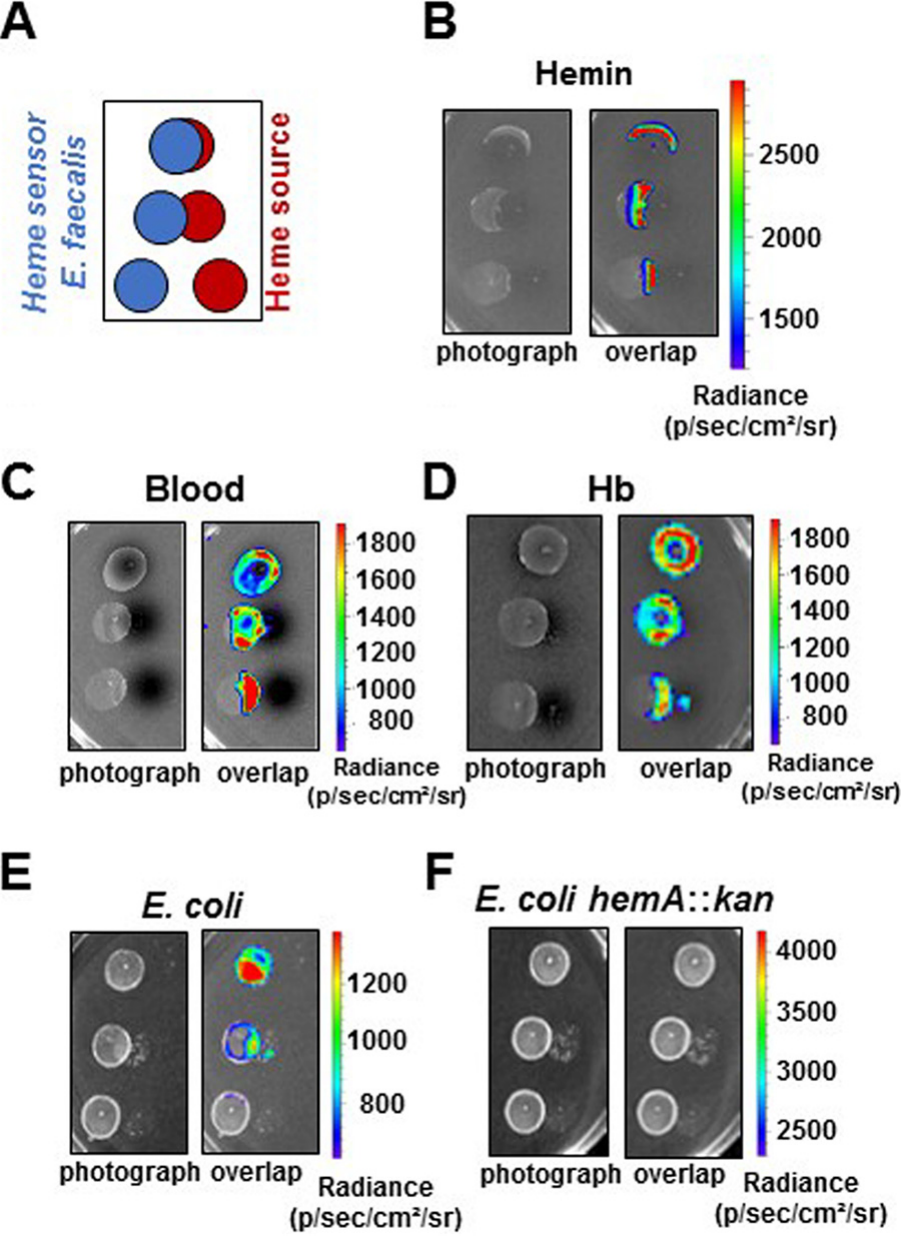
Heme sources for E. faecalis in the GIT. (A) Intracellular heme sensing setup. The indicated heme sources (10 μl) are deposited on M17G solid medium as shown in red. The WT (pP_hrtBA_-*lux*) heme sensor plasmid is plated as 10-μl spots at an OD_600_ of 0.01 as shown in blue. The plates were incubated at 37°C for 16 h and imaged in the IVIS 200 system (acquisition time, 10 min; binning 8). The results are representative of three independent experiments. (B) Visualization of heme sensing from hemin deposits. Hemin (1 mM) in PBS was used as described above for panel A. (C and E) Heme from blood (bovine) and hemoglobin (human) are heme sources for E. faecalis. Heparinized bovine blood (Thermo Fisher) and freshly dissolved human hemoglobin (1 mM) in PBS were used as described above for panel A. (F and G) E. coli is a heme donor for E. faecalis. E. coli (NEB10; New England Biolabs) (F) or a mutant strain that cannot synthesize heme (*hemA*::*kan*) (G) at an OD_600_ of 0.1 were deposited on M17G plates as described above for panel A. Only the heme-synthesizing strain was able to crossfeed heme to E. faecalis. Panels B to F show representative results of three independent experiments.

10.1128/mBio.03392-20.9 FIG S6 Time course of Enterococcus faecalis OG1RF survival in the GIT of mice following ingestion. (A) Female BALB/c mice were force fed with 10^8^ CFU of WT(pP_Ø_-*lux*) (control strain) or WT(p*lux*) (tracking strain). At the indicated times postinoculation, mice were anesthetized with isoflurane and imaged in the IVIS 200 system (acquisition time, 20 min; binning 16). Results are representative of three independent experiments. (B) Human and mouse fecal waters activate heme sensing. Human feces from three healthy human laboratory volunteers (A) and mouse feces from 6-month-old female BALB/c mice (B) were resuspended in PBS (25 μg/ml), and debris was removed by centrifugation at 5,000 × *g* at 4°C for 30 min. Supernatants were sterilely filtered to remove bacteria from the extracts. Fecal water and WT OG1RF (pP_hrtBA_-*lux*) bacteria were plated as described in the legend to Fig. 8. The plates were imaged in the IVIS 200 system (acquisition time, 10 min; binning 8). The figure shows representative results from three independent experiments. Download FIG S6, TIF file, 2.7 MB. Copyright © 2021 Saillant et al. 2021 Saillant et al. This content is distributed under the terms of the Creative Commons Attribution 4.0 International license.

### Heme sources for E. faecalis in the GIT.

The results described above imply that E. faecalis internalizes heme in the intestinal environment to activate FhtR. Thus, an interesting question remains as to the identities of heme sources that are accessible to E. faecalis in the GIT. Normal bleeding (occult blood), exacerbated in intestinal pathologies, as well as food (as meat) are considered main sources of heme within the GIT (the second being excluded in mice) (29–32). We thus visualized the ability of hemoglobin (Hb) or blood deposited on plates as schematized (Fig. 8A) to induce P_hrtBA_ from the heme sensor strain WT(pP_hrtBA_-*lux*) as shown with hemin (Fig. 8B). Similarly, luminescence was induced in proximity of Hb and blood deposits as heme sources (Fig. 8C and D). This result suggests that heme from physiologically available sources is internalized by E. faecalis. Crossfeeding of metabolites, including heme between bacteria, has been reported (29, 33). The possibility that E. faecalis could scavenge heme from intestinal resident heme-synthesizing bacteria, such as E. coli—a phylum that becomes prevalent together with E. faecalis throughout dysbiosis—was evaluated. The WT(pP_hrtBA_-*lux*) heme sensor strain was grown in contact with E. coli (as the heme source) as illustrated in Fig. 8A. Strikingly, induction of luminescence was localized to areas of overlap between the two bacteria (Fig. 8E) and required heme synthesis by E. coli, as no sensing could be detected with a heme-defective *hemA*::*kan* mutant (Fig. 8F). This result suggests that heme synthesized by E. coli is internalized by E. faecalis. Thus, heme crossfeeding between bacterial symbionts in the gut might provide a heme source for E. faecalis. We conclude that the E. faecalis heme sensor is activated by the heme sources available in the GIT.

## DISCUSSION

E. faecalis is a core member of the microbiome, and it is also the cause of a variety of severe infections (34). The central role of heme in reprogramming E. faecalis metabolism and fitness led us to investigate how heme homeostasis is controlled. A novel heme sensor, FhtR, is shown here to regulate heme intracellular homeostasis in E. faecalis. FhtR-heme complexes derepress the *hrtBA_Ef_* operon, leading to HrtBA-mediated management of intracellular heme pools. While expression of HrtBA is a conserved strategy in multiple Gram-positive organisms, E. faecalis appears to be the first example of an opportunistic pathogen where HrtBA is not controlled by the two-component system HssRS. BLAST analysis of FhtR homologs in several Gram-positive bacteria showed that the regulator is present only in enterococci, vagococci, and carnobacteria (see Fig. S7A and S7B in the supplemental material for FhtR alignments and phylogenic tree). FhtR shares no homology with HrtR, a TetR regulator of *hrtRBA* in Lactococcus lactis (19). In contrast to HrtR which autoregulates its own expression, *fhtR* is monocistronic and expressed constitutively, implying that only HrtBA*_Ef_* expression is controlled by heme (19).

10.1128/mBio.03392-20.10 FIG S7 FhtR conservation in Gram-positive bacteria. (A) FhtR orthologs in Gram-positive bacteria. Blast analysis of FhtR shows that the protein is conserved in carnobacteri, enterococci, and vagococci. FhtR AA sequences from Carnobacterium divergens (CDIV41), Carnobacterium maltaromaticum (DSM 20342 NODE_78), Vagococcus lutrae (CCUG39187), Vagococcus entomophilus (DSM 24756,1), Enterococcus faecalis (OG1RF), Vagococcus salmoninarum (NCFB 2777), Enterococcus hirae (ATCC 9790), Enterococcus faecium (ATCC 8459), and Enterococcus avium (ATCC 14025) were aligned using Clustal W. Identical amino acids (*), conserved amino acids (:), and partially conserved amino acids (.) are indicated. (B) Phylogenetic analysis of FhtR amino acid sequences. Predicted FhtR as in panel A were aligned using Clustal W, and evolutionary history was inferred using the neighbor-joining method. The tree is drawn to scale, with bootstrap values (×1,000) shown above the nodes. Download FIG S7, TIF file, 2.8 MB. Copyright © 2021 Saillant et al. 2021 Saillant et al. This content is distributed under the terms of the Creative Commons Attribution 4.0 International license.

We characterized FhtR as a heme binding protein through pentacoordinated ligation of the heme iron, implying a tyrosine. This state of coordination is mostly found in heme receptors that transiently bind heme, such as IsdA, IsdC, and IsdH in S. aureus or HmA in Escherichia coli (35). FhtR blocks *hrtBA_Ef_* transcription by binding to two distinct 14-nt inverted repeats sequences in its promoter region. Alleviation of repression occurs when the heme-FhtR complex loses its affinity for its DNA binding sites. Conformational changes upon ligand binding is a shared mechanism among TetR regulators, leading to uncoupling from DNA (26). We thus hypothesize that these events, which we verified *in vitro*, explain FhtR regulation of the *hrtBA_Ef_* efflux pump in E. faecalis. The unique features of FhtR in E. faecalis compared to other regulators of *hrtBA* genes encoding efflux pumps support the idea that control of HrtBA-mediated heme homeostasis may vary among bacteria as a function of their lifestyle. It is thus tempting to speculate that differences in host niches, and in heme utilization and metabolism, might explain disparities in bacterial heme sensing mechanisms.

Heme efflux by HrtBA is reported as a bacterial detoxification mechanism that prevents intracellular heme overload (8, 14, 16, 17, 19). We showed here that HrtBA induction is required for E. faecalis survival when heme concentrations reached toxic levels (>25 μM). Yet, *hrtBA_Ef_* was induced at heme concentrations as low as 0.1 μM, suggesting that heme efflux is also needed at nontoxic levels. Interestingly, E. faecalis carries a gene that encodes the heme-dependent catalase whose activity relies on the amount of heme in the cytoplasm that is indirectly regulated by FhtR. This enzyme not only binds heme and thus lowers free heme levels, but it also actively lowers oxidative stress generated by heme. It will be of interest to determine the hierarchy of heme binding between FhtR and catalase *in vivo*.

To date, no heme import function has been identified in E. faecalis or in other tested Gram-positive bacteria that cannot synthesize heme (13, 18). BLAST analysis of these bacteria failed to identify genes of the *isd* heme import system described in Staphylococcus aureus (36, 37). In S. aureus, heme receptors and transporters are induced in iron-depleted growth media, and imported heme is used as an iron source (36). Thus, our findings led us to question the need for a dedicated transport system to internalize exogenous heme in E. faecalis and to propose an alternative hypothesis. We noted that HrtBA*_Ef_* is a member of the MacB family of efflux pumps that is distinct from other structurally characterized ABC transporters (38). A model based on MacB transport of antibiotics and antimicrobial peptides in Streptococcus pneumoniae proposed that transmembrane conformational changes promote lateral entry of substrates in the membrane before they reach the cytoplasm (39). On the basis of the previous and present data (23), we propose that HrtB*_Ef_* has the integral role as the heme “gatekeeper” in the cell. Like MacB antibiotics and antimicrobial substrates (40), membrane-bound heme could either enter passively into the intracellular compartment and or be effluxed by HrtB before this step. Altogether, our results place HrtBA*_Ef_* at the forefront of heme homeostasis in E. faecalis that is dependent on the key role of FhtR to adapt to the dichotomy between toxicity and benefits of heme which may be crucial in the host.

*In vivo* bioluminescence imaging of E. faecalis using an FhtR-based sensor identified the GIT as an environment where HrtBA*_Ef_* is expressed. The gut lumen of healthy individuals contains heme, independently of the nature of ingested food or of the microbiota (29–32). Heme in the GIT is reported to mainly originate from Hb from normal bleeding (occult blood) (41). Accordingly, E. faecalis was able to internalize heme from blood and Hb *in vitro*. In addition, a common microbiota constituent, Escherichia coli, is shown to be a heme donor, suggesting a novel basis for intestinal bacterial interactions. As several phyla composing the core microbiota are heme auxotrophs with vital heme requirements, it is tempting to hypothesize that normal or disease-associated fluctuations in host heme levels could be detected by FhtR to adjust its intracellular level and optimize bacterial fitness. Interestingly, E. faecalis causes a variety of severe infections, most often among antibiotic-treated hospitalized patients with intestinal dysbiosis favoring high E. coli and enterobacterial populations (42). It will be interesting to evaluate the impact of HrtBA and FhtR in E. faecalis fitness and virulence in *in vivo* models. Taken together, our results suggest that the FhtR sensor and the HrtBA*_Ef_* heme gatekeeper allow E. faecalis to optimize its adaptation to variable heme pools in the host.

## MATERIALS AND METHODS

### Bacterial strains and growth conditions.

Bacterial strains and plasmids used in this work are listed in Table S1 in the supplemental material. E. coli NEB10 (New England Biolabs) was grown in LB medium, and E. coli C600 *hemA*::*kan* was grown in M17 medium supplemented with 0.5% glucose (M17G). Experiments with E. faecalis were all performed using strain OG1RF and derivatives (Table S1). E. faecalis was grown in static conditions at 37°C in M17G. When needed, antibiotics were used for E. coli at 50 μg · ml^−1^ kanamycin and 100 μg · ml^−1^ ampicillin; for E. faecalis, 30 μg · ml^−1^ erythromycin was used. Oligonucleotides used for plasmid constructions are listed in Table S2. Hemin (Fe-PPIX) (Frontier Scientific) was prepared from a stock solution of 10 mM hemin chloride in 50 mM NaOH. In this report, heme refers to iron protoporphyrin IX regardless of the iron redox state, whereas hemin refers to ferric iron protoporphyrin IX. For growth homogeneity, WT and mutant strains were transformed with the promoterless pTCV-*lac* plasmid compared to complemented strains. Plasmid construction and E. faecalis gene deletion are described in Text S1 in the supplemental material.

10.1128/mBio.03392-20.1 TEXT S1 Supplemental materials and methods. Download Text S1, DOCX file, 0.03 MB. Copyright © 2021 Saillant et al. 2021 Saillant et al. This content is distributed under the terms of the Creative Commons Attribution 4.0 International license.

10.1128/mBio.03392-20.2 TABLE S1 Strains and plasmids. Download Table S1, DOCX file, 0.02 MB. Copyright © 2021 Saillant et al. 2021 Saillant et al. This content is distributed under the terms of the Creative Commons Attribution 4.0 International license.

10.1128/mBio.03392-20.3 TABLE S2 List of oligonucleotides. Download Table S2, DOCX file, 0.01 MB. Copyright © 2021 Saillant et al. 2021 Saillant et al. This content is distributed under the terms of the Creative Commons Attribution 4.0 International license.

### **β**-Galactosidase assays.

Stationary-phase cultures were diluted at an optical density at 600 nm (OD_600_) of 0.01 in M17G and grown to an OD_600_ of 0.5. Hemin was added to cultures, which were further grown for 1.5 h. β-Galactosidase activity was quantified by luminescence in a Spark microplate luminometer (TECAN) using the β-glo substrate (Promega) as described previously (19).

### Cellular ROS quantification.

Stationary-phase cultures were diluted at an OD_600_ of 0.01 in M17G and grown to an OD_600_ of 0.5. Hemin was added to cultures, which were further grown for 1.5 h. Bacteria were washed twice with phosphate-buffered saline (PBS) plus 0.5% glucose by centrifugation at 4°C to remove extracellular heme. Cell pellets were resuspended in PBS plus 0.5% glucose supplemented with 25 μM dihydrorhodamine 123, a cell-permeant fluorescent ROS indicator (Invitrogen). Cell suspensions were distributed into the wells of a 96-well plate. After 15-min incubation, optical density at 600 nm and fluorescence (excitation 500 nm; emission, 536 nm) were measured in a Spark microplate spectrofluorimeter (Tecan).

### Bacterial lysate preparation.

Bacteria were pelleted at 3,500 × *g* for 10 min, resuspended in 20 mM HEPES (pH 7.5) and 300 mM NaCl and disrupted with glass beads (Fastprep; MP Biomedicals). Cell debris was removed by centrifugation at 18,000 × *g* at 4°C for 15 min from the bacterial lysate supernatant. Proteins were quantified by the Lowry method (Bio-Rad).

### Heme concentration determination in bacterial lysates.

Equivalent amounts of proteins (in a volume of 250 μl) were mixed with 250 μl of 0.2 M NaOH, 40% (vol/vol) pyridine, and 500 μM potassium ferricyanide or 5 μl of 0.5 M sodium dithionite (diluted in 0.5 M NaOH), and 500- to 600-nm absorption spectra were recorded in a UV-visible spectrophotometer Libra S22 (Biochrom). Dithionite-reduced minus ferricyanide-oxidized spectra of pyridine hemochromes were used to determine the amount of heme *b* by monitoring the value of the difference between absorbance at 557 nm and 540 nm using a difference extinction coefficient of 23.98 mM^−1^ · cm^−1^ (43).

### Recombinant MBP-FhtR purification.

MBP-FhtR and MBP-FhtR^Y132F^ were purified by affinity chromatography on amylose resin as reported previously (19). Briefly, E. coli NEB10 or C600 Δ*hemA* strains were grown to an OD_600_ of 0.6 or 0.3, respectively, and expression was induced with 1 mM isopropyl-1-thio-β-d-galactopyranoside (IPTG) overnight (ON) at room temperature (RT). Cells were pelleted at 3,500 × *g* for 10 min, resuspended in 20 mM HEPES (pH 7.5) and 300 mM NaCl containing 1 mM EDTA (binding buffer), and disrupted with glass beads (Fastprep; MP Biomedicals). Cell debris was removed by centrifugation at 18,000 × *g* for 15 min at 4°C. MBP-tagged proteins were purified by amylose affinity chromatography (New England Biolabs) following the manufacturer’s recommendations: the soluble fraction was mixed with amylose resin and incubated on a spinning wheel at 4°C for 1 h. The resin was then centrifuged and washed three times with binding buffer. Purified proteins were eluted in binding buffer containing 10 mM maltose and dialyzed against 20 mM HEPES (pH 7.5) and 300 mM NaCl.

### Heme-dependent catalase expression and activity.

KatA expression was monitored on immunoblots with a polyclonal anti-KatA antibody (10). Catalase activity was determined on whole bacteria incubated with 100 μM H_2_O_2_ with the spectrophotometric FOX1 method based on ferrous oxidation in xylenol orange as described previously (44, 45). Absorption was measured at 560 nm.

### Electrophoretic mobility shift assay.

A 325-bp DNA fragment containing the *hrtBA_Ef_* promoter (P_hrtBA_) was amplified by PCR from E. faecalis OG1RF genomic DNA with primer pair (O21-O22) (Table S2). In P_hrtBA_, the two 14-nt palindromic sequences P1 (5′-TTATCAATCGATAA-3′) and P2 (5′-TTATCGATTGATAA-3′) were randomly altered to P1* (5′-ACTTGTATACATAA-3′) and P2* (5′-ATATCTTGTATAAG-3′) to generate three DNA variants P_hrtBA P1*_, P_hrtBA P2*_, and P_hrtBA P1*, P2*_. These fragments were cloned into pUC plasmid (pUC-VS1, pUC-VS2, and pUC-VS3; Table S1) (Proteogenix, France) that were used as templates to PCR amplify the promoter region DNA variants with the primer pairs (O21-O22) (Table S2) that were cloned into pTCV (Table S1). EMSA (electrophoretic mobility shift assay) was performed in 20 mM Tris-HCl (pH 8), 50 mM KCl, 0.2 mM MgCl_2_, 1 mM EDTA, 0.2 mM dithiothreitol (DTT), and 5% glycerol as reported previously (19). Binding was analyzed by gel electrophoresis on a 7% polyacrylamide gel in Tris-borate-EDTA (TBE) buffer stained with GelRed (Biotium) following electrophoresis.

### Ethics statement.

Animal experiments were conducted in strict accordance with the recommendations in the guidelines of the Code for Methods and Welfare Considerations in Behavioural Research with Animals of the EEC council (Directive 2010/63/EU). The protocols were approved by the Animal Care and Use Committee at the Research center of Jouy-en-Josas (COMETHEA; protocol number 15–61) and by the Ministry of Education and Research (APAFIS 2277-2015081917023093 v4). All efforts were undertaken to minimize animal suffering. All experimental procedures were performed in biosafety level 2 facilities.

### *In vivo* heme sensing assay in the mouse GIT.

For inoculation in the digestive tract, E. faecalis strains were prepared as follows. E. faecalis OG1RF precultures were diluted and grown in M17G to an OD_600_ of 0.5 that was determined to correspond to 6 × 10^8^ CFU/ml. Bacteria were then centrifuged at 6,000 rpm at 4°C for 15 min, and pellets were resuspended in PBS to a final concentration of 2 × 10^8^ cells/ml. Bacterial stocks were aliquoted and frozen in liquid nitrogen. Aliquots were kept at −80°C until use. Bacterial counts were confirmed by plating serial dilutions of cultures. Six-week-old female BALB/c mice (Janvier, France) were orally administered by gavage of 10^8^ CFU using a feeding tube. Image acquisition of isoflurane-anesthesized mice was performed at the indicated time following gavage. Following image acquisition, mice were removed from the IVIS 200 imaging system and immediately sacrificed by cervical dislocation. When indicated, the animals were dissected for imaging of the isolated organs. The *in vivo* luminescence imaging procedure is described in Text S1 in the supplemental material.
