# Supplementary figures and images for: A Novel Enterococcus faecalis Heme Transport Regulator (FhtR) Senses Host Heme To Control Its Intracellular Homeostasis
Source: mBio. 2021 Feb 2;12(1):e03392-20. doi: 10.1128/mBio.03392-20 (PMC7858072; doi:10.1128/mBio.03392-20)

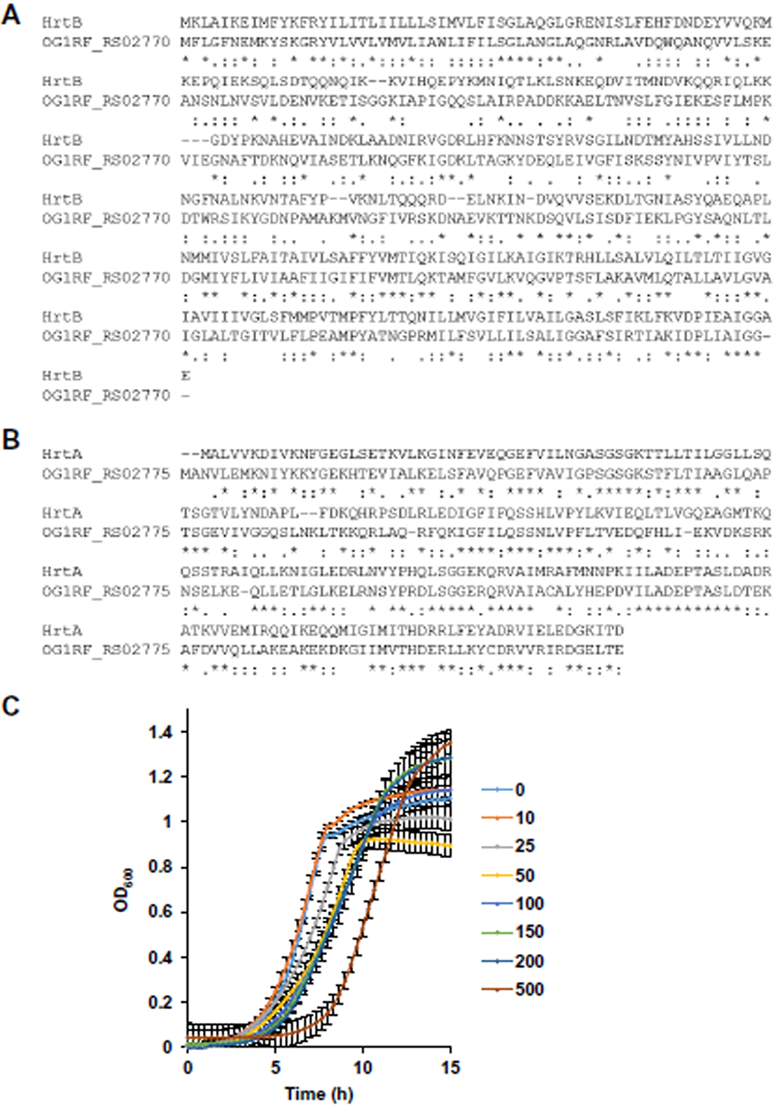

Supplement: FIG S1 [file mBio.03392-20-sf001.tif]

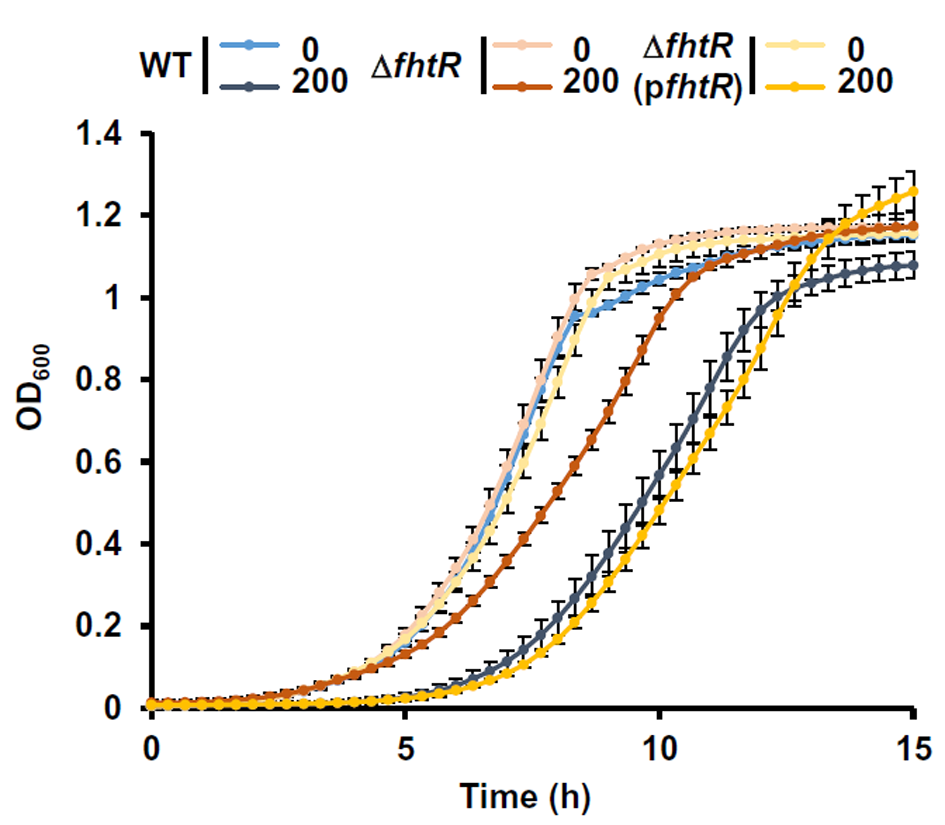

Supplement: FIG S2 [file mBio.03392-20-sf002.tif]

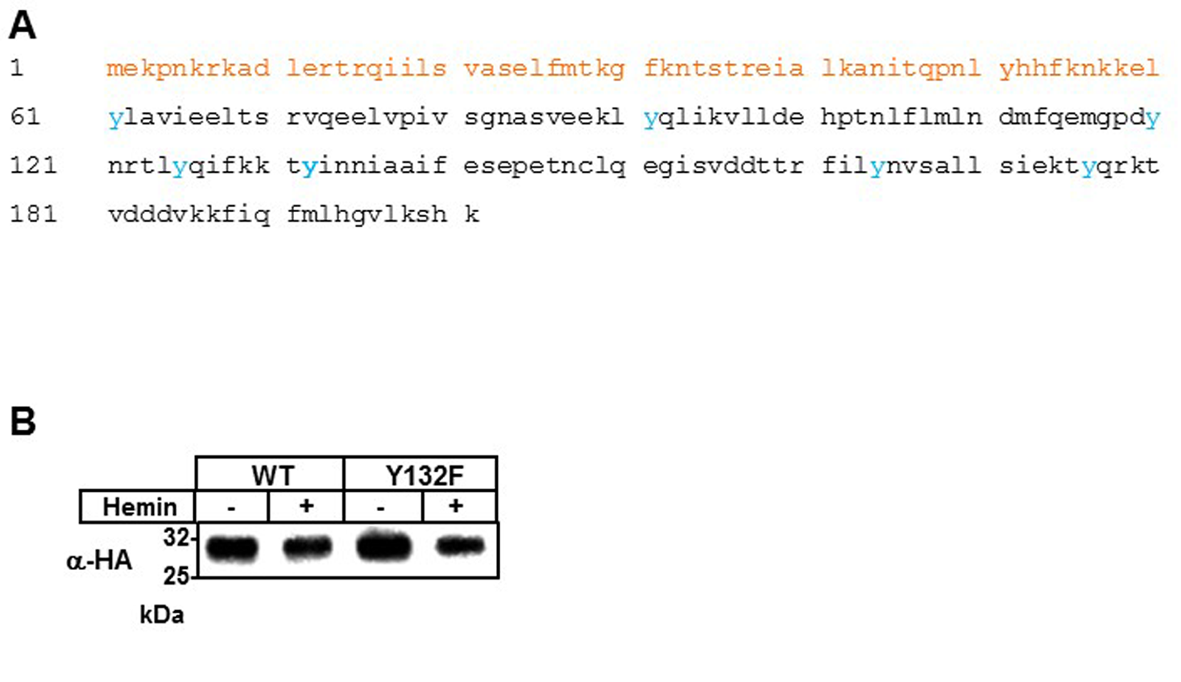

Supplement: FIG S3 [file mBio.03392-20-sf003.tif]

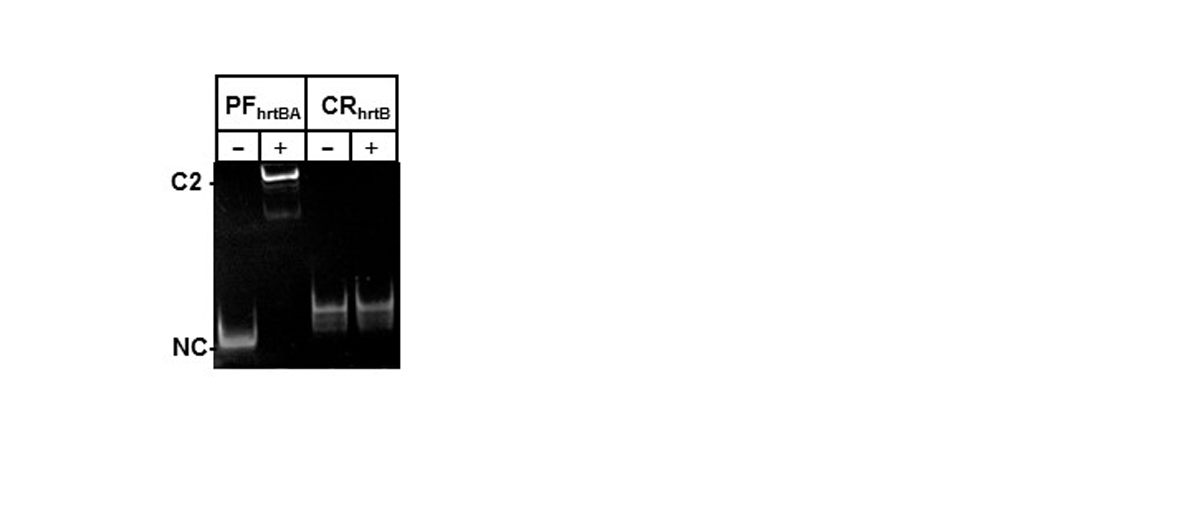

Supplement: FIG S4 [file mBio.03392-20-sf004.tif]

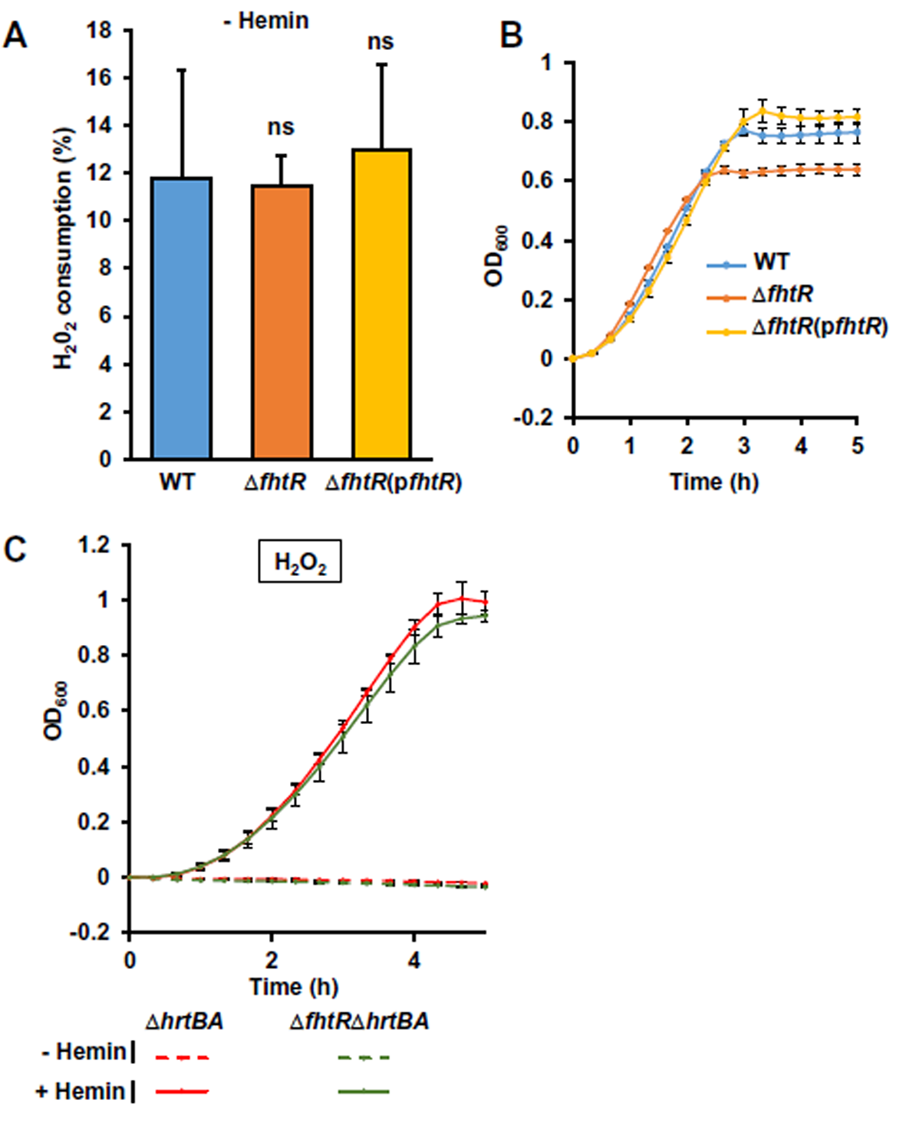

Supplement: FIG S5 [file mBio.03392-20-sf005.tif]

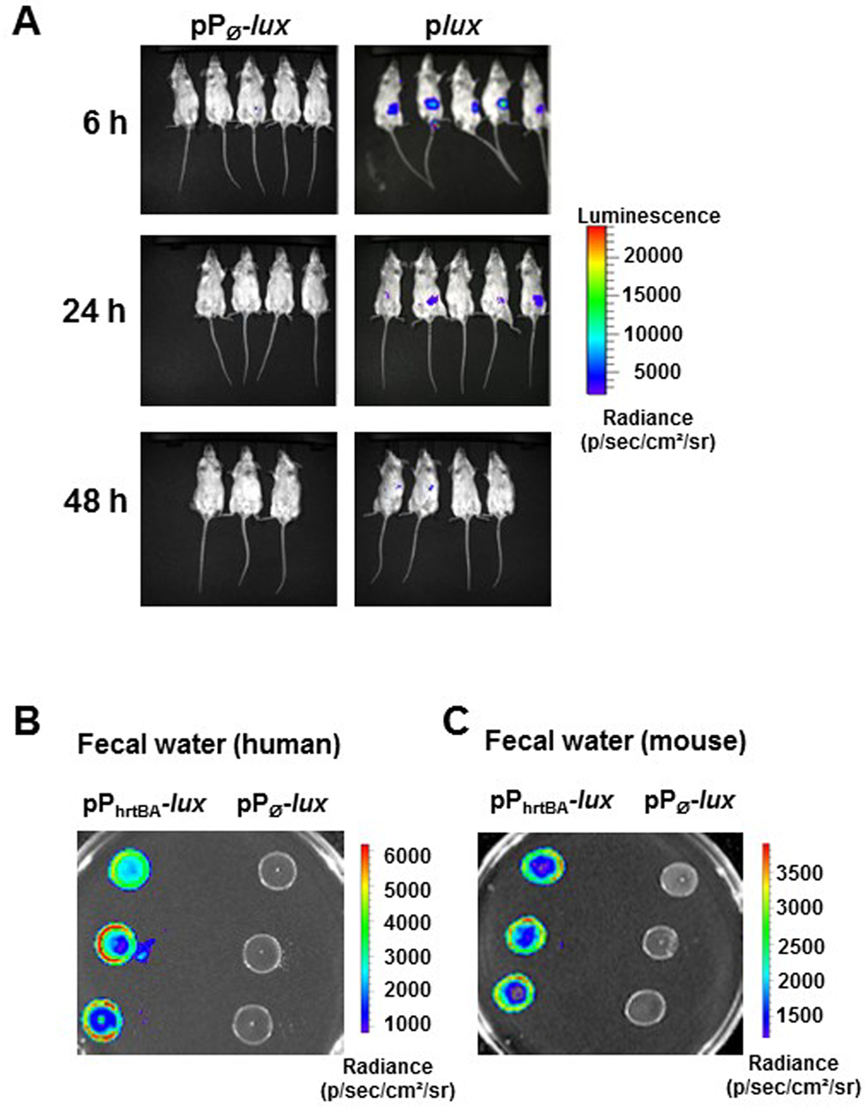

Supplement: FIG S6 [file mBio.03392-20-sf006.tif]

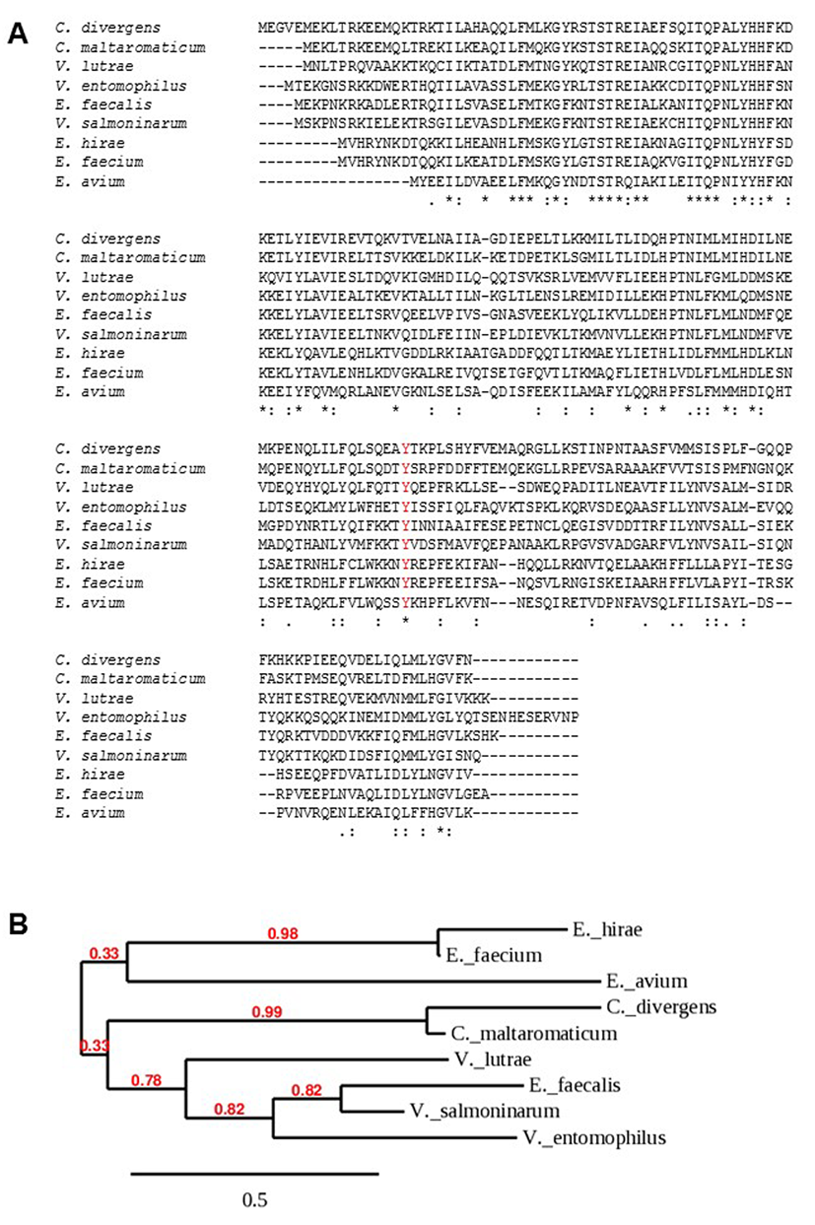

Supplement: FIG S7 [file mBio.03392-20-sf007.tif]
