# Supplementary material for: A Novel Enterococcus faecalis Heme Transport Regulator (FhtR) Senses Host Heme To Control Its Intracellular Homeostasis
Source: mBio. 2021 Feb 2;12(1):e03392-20. doi: 10.1128/mBio.03392-20 (PMC7858072; doi:10.1128/mBio.03392-20)
Supplement: TEXT S1 [file mBio.03392-20-s0001.docx]

**Supplemental materials and methods.**

**Plasmid construction**.The plasmids [1-9] were obtained with similar cloning strategy : PCR amplification of the described DNA inserts with oligonucleotides from Table S2, digestion with (*Eco*RI, *Bam*HI) and ligation with the (*Eco*RI, *Bam*HI) digested plasmid pTCV-*lac* (1) (Table S1). For the following plasmids: [1], pP_hrtBA_-*lac* (pTCV-VS2); [2], p*fhtR* (pP_fhtR_-*fhtR,* P_hrtBA_-*lac*; pTCV-VS3) and [3], pP_fhtR_-*lac* (pTCV-VS4); fragments containing either P_hrtBA,_ P_fhtR_-*fhtR-*P_hrtBA_ or P_fhtR_ DNA sequences were PCR amplified with the primers pairs (O1-O2), (O2-O3) or (O3-O4) respectively. Plasmid [4], p*fhtR*-HA (pP_fhtR_-*fhtR-*HA*,* P_hrtBA_-*lac*; pTCV-VS5) was obtained by PCR amplification of 2 DNA fragments, P_fhtR_-*fhtR* and P_hrtBA_ using primer pairs (O3-O5) and (O2-O6), respectively, followed by their fusion by PCR-overlap with the primers (O2-O3) leading to the addition of nt encoding the hemagglutinin influenza epitope (HA, YPYDVPDYA) at the 3’ end of *fhtR* (2). The mutation Y132F was introduced into the coding sequence of *fhtR* in the plasmids [5], pP_fhtR_-*fhtR*^Y132F^ (pTCV-VS6) and [6], pP_fhtR_-*fhtR*^Y132F^-HA (pTCV-VS7) by a PCR-overlap with (O2-O3) of 2 PCR products obtained with primer pairs (O3-O7) and (O2-O8) using pTCV-VS3 and pTCV-VS5 as templates, respectively. Plasmids [7], pP_hrtBA P1*_-*lac* (pTCV-VS8); [8], pP_hrtBA P2*_-*lac* (pTCV-VS9) and [9], pP_hrtBA P1* P2*_-*lac* (pTCV-VS10 ) were obtained by PCR amplification of P_hrtBA_ nt sequence with primers pair (O1-O2) using pUC-VS1, pUC-VS2 and pUC-VS3 as templates, respectively (Table S1). The plasmid pP_hrtBA_-*lux* (pTCV-VS11) was constructed as described for pP_hrtBA_-*lac* using the plasmid vector pTCV-J22 (Table S1). Plasmids pMBP-FhtR and pMBP-FhtR^Y132F^ (Table S1) were obtained by PCR amplification of FhtR ORF from *E. faecalis* OG1RF genomic DNA and pP_fhtR_-*fhtR*^Y132F^ respectively, with the primers pairs (O9-O10) (Table S2). The resulting fragments were digested with *Eco*RI and *Pst*I and ligated with the plasmid pMAL-c4X (New England Biolabs) (Table S1). The 3 plasmids p∆*hrtBA_Ef_* (pG1-VS1), p∆*fhtR* (pG1-VS2) and p∆*fhtR*∆*hrtBA_Ef_* (pG1-VS3) were constructed by PCR amplification of 2 fragments of ~ 600-750 pb flanking the genes OG1RF_RS02770-OG1RF_RS02775 (*hrtBA_Ef_*); OG1RF_RS02765 (*fhtR*) gene and OG1RF_RS02765-OG1RF_RS02775 (*fhtR*-*hrtBA_Ef_*) with the respective (O11-O12) and (O13-O14); (O15-O16) and (O17-O18); (O15-O19) and (O14-O20) oligonucleotides (Table S1 and S2). Each pair of fragments was fused by PCR overlap as above with the DNA primers pairs (O11-O13), (O15-O18) and (O14-O15), respectively (Table S2). The resulting fragments were digested with *Bam*HI and *Hind*III and ligated into the thermosensitive pG1 (pG1-VS2, Table S1). All plasmids were verified by DNA sequencing.

***E. faecalis* Δ*hrtBA_Ef_*, Δ*fhtR* and Δ*fhtR*Δ*hrtBA_Ef_* mutants.** The plasmids p∆*hrtBA_Ef_* (pG1-VS1), p∆*fhtR* (pG1-VS2) and p∆*fhtR*∆*hrtBA_Ef_* (pG1-VS3) (Table S1) were transformed by electroporation in *E. faecalis* OG1RF strain. The double cross-over events leading to ∆*hrtBA_Ef_,* ∆*fhtR and* mutants and ∆*fhtR*∆*hrtBA_Ef_* double mutant were obtained as described (3). Correct inactivation of the targeted genes was confirmed by DNA sequencing.

**Titration of MBP-FhtR with hemin.** *ApoFhtR: hemin stoichiometry*. Hemin binding affinity of MBP-FhtR was determined by adding 0.5 to 1 μl increments of a 200 μM hemin solution to cuvettes containing 100 μl test samples of 20 μM MBP-FhtR in 20 mM Hepes pH 7.5, pH 8, 300 mM NaCl, or reference sample without protein. Spectra were measured from 300 nm to 700 nm in a UV-visible spectrophotometer Libra S22 (Biochrom). OD_407_ was plotted against hemin concentration and data were fitted to a one-binding site model (with a calculated extinction coefficient of bound hemin of ε_407_ = 70 mM^-1^.cm^-1^) as described (9). *Hemin binding affinity*. Tryptophan fluorescence quenching (emission: from 300 nm to 400 nm; excitation: 280 nm) was determined as a function of hemin concentration, using a Carry Eclipsefluorimeter. Curves Absorbance and fluorescence curves fitting were fitted to the one-binding site model with the following equation:

$$\mathrm{OD}_{407nm} =a_{0}+\Delta A\times\left[ PL \right]=a_{0}+\Delta A\times0.5(K_{d}+[P_{0}]+[L_{0}]-\sqrt{{(K_{d} +[P_{0}]+[L_{0}])}^{2}-4[P_{0}][L_{0}])}$$

$$F =a_{0}-\Delta F\times[PL]=a_{O}-\Delta F\times0.5(K_{d}+[P_{0}]+[L_{0}]-\sqrt{{(K_{d} +[P_{0}]+[L_{0}])}^{2}-4[P_{0}][L_{0}])}$$

Here, OD_407nm_ and F are respectively the absorbance and fluorescence amplitudes. ΔA and ΔF are the normalized amplitudes of the saturated absorbance and quenching, respectively. [PL] is the concentration of liganded protein, [P_0_] and [L_0_] are the concentrations of the total protein and ligand, respectively and a_0_ is the starting absorbance/fluorescence. Fitting of this equation was was done with the non-linear regression function of GraphPad Prism 7 software.

**EPR spectroscopy.** X-band cw-EPR spectra were recorded with a Bruker Elexsys 500 X-band spectrometer equipped with a standard ER 4102 (Bruker) X-band resonator, a Bruker teslameter, an Oxford Instruments cryostat (ESR 900) and an Oxford ITC504 temperature controller. The spectra shown were recorded at 15 K with a modulation frequency equal to 100 kHz, a modulation amplitude equal to 25 gauss, a microwave power equal to 5 mW and a microwave frequency equal to 9.49 GHz.

**Luminescence imaging.** Light emission from whole animals, organs, feces or plates was measured in an In Vivo Imaging system (IVIS 200, Caliper Life Sciences, USA) equipped with the Living image software (version 4.0, Caliper Life Science, USA) as reported previously (4). Mice were anesthetized during imaging via inhalation of isoflurane. For each acquisition, a grayscale photographic and a pseudocolor luminescence images were taken. Bioluminescence images were acquired with a 25 cm field of view (FOV), medium (8) or large (16) binning factor and an exposure time as indicated in the figure legends. A digital false-color photon emission image was generated according to photon counts within a constant region of interest (ROI) corresponding to the surface of the entire surface of the image. Rainbow images show the relative level of luminescence ranging from low (blue), to medium (green), to high (yellow/red). Photon emission was measured in radiance (photon per second per square centimeter per steradian, p.s^-1^.cm^-2^_._sr^-1^). Luminescence images were adjusted for color scale (minimum and maximum) and corrected (final pixel size: binning 8; pixel size smoothing: 3x3) with the Living Image software. Photographic and overlay images were adjusted for brightness and contrast using PhotoShop CS3 (Adobe Systems, San Jose, CA).

**References**

1. Poyart C, Trieu-Cuot P. 1997. A broad-host-range mobilizable shuttle vector for the construction of transcriptional fusions to B-galactosidase in Gram-positive bacteria. FEMS Microbiology Letters 156:193-198.

2. Lechardeur D, Fernandez A, Robert B, Gaudu P, Trieu-Cuot P, Lamberet G, Gruss A. 2010. The 2-Cys peroxiredoxin alkyl hydroperoxide reductase c binds heme and participates in its intracellular availability in *Streptococcus agalactiae*. J Biol Chem 285:16032-16041.

3. Maguin E, Prevost H, Ehrlich SD, Gruss A. 1996. Efficient insertional mutagenesis in lactococci and other gram-positive bacteria. J Bacteriol 178:931-5.

4. Joubert L, Dagieu JB, Fernandez A, Derre-Bobillot A, Borezee-Durant E, Fleurot I, Gruss A, Lechardeur D. 2017. Visualization of the role of host heme on the virulence of the heme auxotroph *Streptococcus agalactiae*. Sci Rep 7:40435.
