# Supplementary material for: A Novel Enterococcus faecalis Heme Transport Regulator (FhtR) Senses Host Heme To Control Its Intracellular Homeostasis
Source: mBio. 2021 Feb 2;12(1):e03392-20. doi: 10.1128/mBio.03392-20 (PMC7858072; doi:10.1128/mBio.03392-20)
Supplement: TABLE S1 [file mBio.03392-20-st001.docx]

**Table S1.** Strains and plasmids.

| ***Strain/plasmid*** | ***Characteristics*** | ***Source/ reference*** |
| --- | --- | --- |
| ***Strain***  ***E. coli*** | | |
| NEB 10 | Δ*(ara-leu) 7697 araD139 fhuA* Δ*lacX74 galK16 galE15 e14-* ϕ*80*d*lacZ*Δ*M15 recA1 relA1 endA1 nupG rpsL* (StrR) *rph spoT1* Δ*(mrr-hsdRMS-mcrBC)* | New England Biolabs |
| C600 *hemA*::*kan* | *hemA*::*kan*, derivative of C600, Kan^R^. | (1) |
| ***E. faecalis*** | | |
| OG1RF | Strain OG1RF rifampicin and fusidic acid resistant derivative of OG1, a human isolate. | (2) |
| OG1RFVS1 | OG1RF ∆*hrtBA_Ef_*, deletion of *hrtB* and *hrtA* genes. | This study |
| OG1RFVS2 | OG1RF ∆*fhtR*, deletion of *fhtR* gene. | This study |
| OG1RFVS3 | OG1RF ∆*fhtRhrtBA_Ef_*, deletion of *fhtR, hrtB* and *hrtA* genes. | This study |
| EMB1 | *katA*::*ISS1,*derivative of OG1RF, Tet^R^. | (3) |
| ***Plasmid*** | | |
| pTCV-*lac* | Conjugative *E. coli* Gram positive bacteria shuttle plasmid carrying the promoterless *E. coli lacZ* gene for constructing transcriptional fusions. Ery^R^, Kan^R^. | (4) |
| pMAL-c4X | Cloning vector for expression of MBP tagged proteins in *E. coli*. Amp^R^. | New England Biolabs |
| pG1 | Temperature sensitive cloning vector. Amp^R^, Ery^R^. | (5) |
| PG1-VS1 | p∆*hrtBA_Ef_, hrtBA_Ef_* fragment cloned into pG1 to obtain the ∆*hrtBA_Ef_* mutant. | This study |
| pG1-VS2 | p∆*fhtR. fhtR* fragment cloned into pG1 to obtain ∆*fhtR* mutant. | This study |
| pG1-VS3 | p∆*fhtRhrtBA_Ef_*. *fhtRhrtBA_Ef_* fragment cloned into pG1 to obtain the ∆*fhtR*∆*hrtBA_Ef_* mutant. | This study |
| pMBP-FhtR | Expression of N-terminal MBP-tagged *E. faecalis* FhtR. Cloned into pMAL-c4X. | This study |
| pMBP-FhtR^Y132F^ | Expression of N-terminal MBP-tagged *E. faecalis* FhtR^Y132F^. Cloned into pMAL-c4X. | This study |
| pTCV-VS1 | pP_hrt_-*hrtR-lac*, DNA fragment containing the promoter region and *hrtR* cloned in pTCV-*lac*. | (6) |
| pTCV-VS2 | pP_hrtBA_-*lac*, *E. faecalis*  OG1RF *hrtBA_Ef_* promoter region cloned into pTCV-*lac.* | This study |
| pTCV-VS3 | p*fhtR*: pP_fhtR_-*fhtR,* P_hrtBA_-*lac*. *E. faecalis*  OG1RF *fhtR* promoter and gene region and *hrtBA_Ef_* promoter region cloned into pTCV-*lac.* | This study |
| pTCV-VS4 | pP_fhtR_-*lac*. *E. faecalis*  OG1RF *fhtR* promoter region cloned into pTCV-*lac.* | This study |
| pTCV-VS5 | p*fhtR*-HA: pP_fhtR_-*fhtR-HA,* P_hrtBA_-*lac*. *E. faecalis*  OG1RF *fhtR* promoter, Ct HA-tagged *fhtR* gene region and *hrtBA_Ef_* promoter region cloned into pTCV-*lac.* | This study |
| pTCV-VS6 | p*fhtR*^Y132F^ : pP_fhtR_-*fhtR*^Y132F^*,* P_hrtBA_-*lac*, *E. faecalis*  OG1RF *fhtR* promoter, *fhtR*^Y132F^ gene region and *hrtBA* promoter region cloned into pTCV-*lac.* | This study |
| pTCV-VS7 | p*fhtR*^Y132F^-HA : pP_fhtR_-*fhtR*^Y132F^-HA *,* P_hrtBA_-*lac*. *E. faecalis*  OG1RF *fhtR* promoter, Ct HA-tagged *fhtR*^Y132F^ region and *hrtBA_Ef_* promoter region cloned into pTCV-*lac.* | This study |
| pTCV-VS8 | pP_hrtBA P1*_-*lac*. *E. faecalis* OG1RF *hrtBA_Ef_* promoter with 14-nt palindromic sequence P1, TTATCAATCGATAA replaced by ACTTGTATACATAA cloned into pTCV-*lac.* | This study |
| pTCV-VS9 | pP_hrtBA P2*_-*lac*. *E. faecalis* OG1RF *hrtBA_Ef_* promoter with 14-nt palindromic sequence P2, TTATCGATTGATAA replaced by ATATCTTGTATAAG cloned into pTCV-*lac.* | This study |
| pTCV-VS10 | pP_hrtBA P1*, P2*_-*lac*. *E. faecalis*  OG1RF *hrtBA_Ef_* promoter which P1 and P2 palindromic sequence have been replaced cloned into pTCV-*lac.* | This study |
| pTCV-J22 | pP_Ø_-*lux*. *luxABCDE* reporter genes. | (7) |
| pTCV-VS11 | pP_hrtBA_-*lux*. *E. faecalis*  OG1RF *hrtBA_Ef_* promoter region cloned into pTCV-J22*.* | This study |
| pTCV-J24 | P*lux*. P_23_ promoter cloned in pTCV-J22. | (7) |
| pUC-VS1 | A 325-bp DNA fragment containing pP_hrtBA P1*_ cloned in pUC57. Amp^R^. | Proteogenix |
| pUC-VS2 | A 325-bp DNA fragment containing pP_hrtBA P2*_ cloned in pUC57. Amp^R^. | Proteogenix |
| pUC-VS3 | A 325-bp DNA fragment containing pP_hrtBA P1*, P2*_ in pUC57. Amp^R^. | Proteogenix |

**References**

1. Letoffe S, Delepelaire P, Wandersman C. 2006. The housekeeping dipeptide permease is the *Escherichia coli* heme transporter and functions with two optional peptide binding proteins. Proc Natl Acad Sci U S A 103:12891-6.

2. Dunny GM, Brown BL, Clewell DB. 1978. Induced cell aggregation and mating in *Streptococcus faecalis*: evidence for a bacterial sex pheromone. Proc Natl Acad Sci U S A 75:3479-83.

3. Baureder M, Hederstedt L. 2012. Genes important for catalase activity in *Enterococcus faecalis*. PLoS One 7:e36725.

4. Poyart C, Trieu-Cuot P. 1997. A broad-host-range mobilizable shuttle vector for the construction of transcriptional fusions to B-galactosidase in Gram-positive bacteria. FEMS Microbiology Letters 156:193-198.

5. Mistou MY, Dramsi S, Brega S, Poyart C, Trieu-Cuot P. 2009. Molecular dissection of the secA2 locus of group B Streptococcus reveals that glycosylation of the Srr1 LPXTG protein is required for full virulence. J Bacteriol 191:4195-206.

6. Lechardeur D, Cesselin B, Liebl U, Vos MH, Fernandez A, Brun C, Gruss A, Gaudu P. 2012. Discovery of an intracellular heme-binding protein, HrtR, that controls heme-efflux by the conserved HrtB HrtA transporter in *Lactococcus lactis*. J Biol Chem 287:4752-4758.

7. Joubert L, Dagieu JB, Fernandez A, Derre-Bobillot A, Borezee-Durant E, Fleurot I, Gruss A, Lechardeur D. 2017. Visualization of the role of host heme on the virulence of the heme auxotroph *Streptococcus agalactiae*. Sci Rep 7:40435.
