# Supplementary material for: A Novel Enterococcus faecalis Heme Transport Regulator (FhtR) Senses Host Heme To Control Its Intracellular Homeostasis
Source: mBio. 2021 Feb 2;12(1):e03392-20. doi: 10.1128/mBio.03392-20 (PMC7858072; doi:10.1128/mBio.03392-20)
Supplement: TABLE S2 [file mBio.03392-20-st002.docx]

**Table S2.** List of oligonucleotides.

| **Primer** | **Sequence 5’→ 3’** | **Target** |
| --- | --- | --- |
| **O1** | CCGGAATTCCCAGCGAAAAACAGTGGATGACGAT | P_hrtBA_ |
| **O2** | ATTAATGGATCCGAATGGTTCACCTCTTTCTTC | P_hrtBA_ |
| **O3** | TAATAAGAATTCTTTTTATATGGACTGCAGTGG | P_fhtR_ |
| **O4** | TTATTAGGATCCGGTTTTCTCGCCCCCTGC | P_fhtR_ |
| **O5** | ACAGGGCTTCTCTATTTTTTAAGCATAATCTGGAACATCATATGGATACA | *fhtR-HA** |
| **O6** | GCGTTTTGAAAAGTCACAAAATGTATCCATATGATGTTCCAGATTATGCT | *fhtR-HA** |
| **O7** | CGCAATATTATTGATGAAGGTTTTTTTGAAAAT | *Y132F* |
| **O8** | ATTTTCAAAAAAACCTTCATCAATAATATTGCG | *Y132F* |
| **O9** | TAATAAGAATTCATGGAAAAGCCAAATAAACG | *MBP-fhtR* |
| **O10** | TTATTACTGCAGTTATTTGTGACTTTTCAAAA | *MBP-fhtR* |
| **O11** | CGGGGTACCGCTGATTTAGAACGTACACGGC | Δ*hrtBA_Ef_* |
| **O12** | GATCATGTTCTCTTTTATTTGTTTTGCATGAATGGTTCACCTCTTTCTTCT | Δ*hrtBA_Ef_* |
| **O13** | AGAAGAAAGAGGTGAACCATTCATGCAAAACAAATAAAAGAGAACATGATC | Δ*hrtBA_Ef_* |
| **O14** | AAAACTGCAGGCGATTGATACGGAAATGAAACGCC | Δ*hrtBA_Ef_* |
| **O15** | TAATTAAAGCTTACGAAGCAATTAAAGGGCGC | Δ*fhtR* |
| **O16** | CAACAGGGCTTCTCTATTTTGGTTTTCTCGCCCCCTGCTG | Δ*fhtR* |
| **O17** | CAGCAGGGGGCGAGAAAACCAAAATAGAGAAGCCCTGTTG | Δ*fhtR* |
| **O18** | ATGCGGATCCCCCTGCAGTTAATTTGTCGCC | Δ*fhtR* |
| **O19** | CTTTTATTTGTTTTGGGTTTTCTCGCCCCC | Δ*fhtR*Δ*hrtBA_Ef_* |
| **O20** | GGGGGCGAGAAAACCCAAAACAAATAAAAGAGAACATGATCGG | Δ*fhtR*Δ*hrtBA_Ef_* |
| **O21** | CCAGCGAAAAACAGTGGATGACGAT | PF_hrtBA_ |
| **O22** | CGCTAAACCTGACAAAATAAAAATTAACC | PF_hrtBA_ |
| **O23** | GGCCAATAGTAATTTAAATGTATCAGTGTT | CR*_hrtB_* |
| **O24** | ACTTTTAGAAATAAAGCCGACAATT | CR*_hrtB_* |

The restriction sites included in oligonucleotides are underlined. * HA sequence corresponds to the peptide MYPYDVPDYA.
